# Phosphoproteome and Transcriptome of RA-Responsive and RA-Resistant Breast Cancer Cell Lines

**DOI:** 10.1371/journal.pone.0157290

**Published:** 2016-06-30

**Authors:** Marilyn Carrier, Mathilde Joint, Régis Lutzing, Adeline Page, Cécile Rochette-Egly

**Affiliations:** 1 Department of Functional Genomics and Cancer, IGBMC (Institut de Génétique et de Biologie Moléculaire et Cellulaire), INSERM, U964, CNRS, UMR7104, Université de Strasbourg, 1 rue Laurent Fries, BP 10142, 67404 Illkirch Cedex, Strasbourg, France; 2 Proteomics Platform, IGBMC (Institut de Génétique et de Biologie Moléculaire et Cellulaire), INSERM, U964, CNRS, UMR7104, Université de Strasbourg, 1 rue Laurent Fries, BP 10142, 67404 Illkirch Cedex, Strasbourg, France; University of Padova, ITALY

## Abstract

Retinoic acid (RA), the main active vitamin A metabolite, controls multiple biological processes such as cell proliferation and differentiation through genomic programs and kinase cascades activation. Due to these properties, RA has proven anti-cancer capacity. Several breast cancer cells respond to the antiproliferative effects of RA, while others are RA-resistant. However, the overall signaling and transcriptional pathways that are altered in such cells have not been elucidated. Here, in a large-scale analysis of the phosphoproteins and in a genome-wide analysis of the RA-regulated genes, we compared two human breast cancer cell lines, a RA-responsive one, the MCF7 cell line, and a RA-resistant one, the BT474 cell line, which depicts several alterations of the “kinome”. Using high-resolution nano-LC-LTQ-Orbitrap mass spectrometry associated to phosphopeptide enrichment, we found that several proteins involved in signaling and in transcription, are differentially phosphorylated before and after RA addition. The paradigm of these proteins is the RA receptor α (RARα), which was phosphorylated in MCF7 cells but not in BT474 cells after RA addition. The panel of the RA-regulated genes was also different. Overall our results indicate that RA resistance might correlate with the deregulation of the phosphoproteome with consequences on gene expression.

## Introduction

Retinoic Acid (RA), the major active derivative of vitamin A, is essential for all steps of life, from the embryo to the adult, through the regulation of the expression of a battery of target genes involved in cell differentiation, proliferation, adhesion, migration, death or survival [1, 2]. These effects of RA are mediated by nuclear receptors, RAR (α, β and γ), which are ligand-dependent regulators of transcription and bind specific response elements (RAREs) located in the promoters of their target genes [1, 3]. Recently, genome-wide high throughput sequencing and chromatin immunoprecipitation coupled with deep sequencing expanded the repertoire of the RA-target genes in several cell lines [3–7]. However, today it is clear that RA also has non-transcriptional effects and activates kinase cascades [8, 9]. These kinases phosphorylate several targets in the cytosol and translocate into the nucleus where they phosphorylate RARs themselves as well as other proteins [8, 10]. Phosphorylation is a widely used mechanism of post-translational modification that controls protein activity, stability, turnover, and interaction with DNA or partner proteins [11].

Cancer with aberrant cell growth and differentiation blockage often results from alterations of the RA pathway and reciprocally, RA has proven anti-cancer capacity due to its ability to induce growth arrest and cell death and to restore cell differentiation [12]. A vast literature reports that several breast cancer cells are sensitive to the antiproliferative activity of RA while others are resistant. Today, there is increasing evidence that cancer, including breast cancer often results from alterations of the signaling pathways [13]. However the overall phosphorylation events and transcriptional pathways that are altered in such RA-resistant cells have not been the object of systematic studies. In fact, most of the RA-induced phosphorylation events are still unknown, making their large-scale analysis instrumental in understanding the complex signaling events initiated by RA.

The purpose of the present study was to determine whether the phosphorylation events induced by RA in RA responsive cells, were altered in RA resistant cells subsequently to alterations of the « kinome ». Therefore we compared two human breast cancer cell lines, the MCF7 cell line, which responds to the antiproliferative action of RA and the BT474 cell line, which is RA-resistant. BT474 cells depict several PI3K mutations and amplification of the erb-b2 receptor tyrosine kinase (*ERBB2*) gene [14], but RA resistance has been correlated to *ERBB2* amplification and to the subsequent alterations of the downstream PIEK/Akt signaling pathway [15]. For both cell lines, the phosphorylated proteins were large-scale analyzed before and after RA treatment, using high-resolution nano-LC-LTQ-Orbitrap mass spectrometry [16, 17] associated to phosphopeptide enrichment [18, 19]. As phosphorylation of RARs and their coregulators is well known to control the expression of RA target genes [20–22], these two cell lines were also compared in a genome-wide analysis of the RA-regulated genes. This study revealed major differences not only in the basal “phosphoproteome” but also in the RA-induced phosphorylation events indicating that alterations of the “kinome” interfere with the ability of RA to activate phosphorylation cascades. Consequently the phosphorylation of several transcription factors including RARα was affected with consequences on RA target genes regulation.

## Materials and Methods

### Cell culture, extracts preparation and digestion for phosphoproteins analysis

MCF-7 and BT474 human breast cancer cell lines were purchased from the American Type Culture Collection (ATCC) and cultured as monolayers under standard conditions as previously described [9, 22]. 17x10^6^ MCF7 or 42x10^6^ BT474 cells were seeded in 15 cm Petri dishes (4 dishes per cell line). When cells were 80–90% confluent, *all-trans* RA (10^−6^ M) (Sigma Aldrich) was added to two dishes and vehicle (0.1% ethanol) to the two others, after 24h in low (1%) serum medium conditions, without insulin and phenol red. Then the cells were scrapped for cytosolic and nuclear extracts preparation.

Cytosolic extracts were obtained by lysis in hypotonic buffer (10 mM HEPES-KOH pH 7.9, 1.5 mM MgCl_2_, 10 mM KCl, 1 mM EDTA and 0.5% Igepal CA-630). Nuclear extracts were obtained by incubating the remaining pellet in extraction buffer (20 mM HEPES-KOH pH 7.9, 1.5 mM MgCl_2_, 420 mM NaCl, 1 mM EDTA and 25% glycerol). Purity of the extracts was assessed by immunoblotting with antibodies against β-tubulin (IGBMC facility) and lamin A/C (Santa Cruz Biotechnology), which are present exclusively in the cytosol and in the nucleus respectively. All buffers were ice-cold and supplemented with 1 mM PMSF, 50 μM NaF, 5 mM Na_3_VO_4_ (Sigma Aldrich), complete protease inhibitor cocktail (PIC) and PhosSTOP (Roche Diagnostics).

Under these conditions, MCF7 cells yielded 3.5 mg of cytosolic proteins and 0.8 mg of nuclear proteins, while BT474 cells yielded 6.5 and 1.6 mg of cytosolic and nuclear proteins respectively.

Only 0.35 mg of each extract preparation were used since it was the maximum analyzable sample size for MS. Samples were TCA-precipitated (12h at 4°C) and centrifuged (14000 rpm, 30 min, 4°C). Pellets were washed twice with 500 μL cold acetone, centrifuged (14000 rpm, 10 min, 4°C), urea-denatured (8M urea in Tris-HCl 0.1 mM), reduced with 5 mM Tris (2-carboxyethyl) phosphine (TCEP, Hampton research) for 30 min, and then alkylated with 10 mM iodoacetamide (30 min in the dark). Reduction and alkylation were performed at room temperature under agitation (850 rpm).

Then the samples were divided in two. One half (175 μg) was digested at 37°C with endoproteinase Lys-C (Wako chemicals) in 8 M urea for 6h, followed by an overnight digestion with modified Trypsin (Promega) in 2 M urea. The other half was digested overnight with Chymotrypsin (Promega) in 1 M urea at 25°C. All enzymes were diluted 1:100 (w/w). The resulting peptides were desalted on C18 spin-columns (Harvard apparatus) and dried (Speed-Vacuum). An aliquot (10 μg) of the Lys-C/Trypsin digest was kept for direct analysis, and the other samples were submitted to phospho-enrichment.

### RARα immunoprecipitation and digestion

In this case, larger amounts of proteins and RARα enrichment by immunoprecipitation were required due to the low abundance of the RARα protein. Therefore, the cells were first amplified as adherent cultures in 15 cm cell culture dishes (MCF7: 60 dishes, BT474 76 dishes). Five days before the experiments, the cells were trypsinized, pooled, counted and divided into two equal 3 L suspension cultures per cell line (MCF7: 2,58 X 10^8^, BT474: 13,8 X 10^8^ cells per 3 L culture). Then cells were treated with 10^−6^ M RA or vehicle for 30 minutes (one 3 L culture per condition) after 24h in low serum medium conditions.

Whole cell extracts were prepared by suspending the cells in lysis buffer (50 mM Tris-HCl pH 7.5, 150 mM NaCl, 5% glycerol and 0.5% Igepal CA-630) supplemented with 1 mM PMSF, 50 μM NaF, 5 mM Na_3_VO_4_, 125 nM okadaic acid (Calbiochem), complete PIC and PhosSTOP. After centrifugation, this protocol yielded around 100 mg proteins per culture.

The whole extracts were incubated with 2 mouse monoclonal antibodies raised against RARα, one recognizing the C-terminal region [Ab9α (F)] and the other one the N-terminal region [Ab10α (A1)] [23], bound to Dynabeads Protein G (Invitrogen). These antibodies raised in house were purified and concentrated from ascitic fluid on protein G sepharose and were excellent for the immunoprecipitation of RARα and its detection by MS. Elution was performed in Protein LoBind Tubes (Eppendorf) with 62.5 mM Tris-HCl pH 6.8 containing 2% SDS, 10% glycerol and 3% β-mercaptoethanol, followed by pH neutralization with Tris-HCl pH 9. The eluates (around 200μg proteins) were reduced, alkylated (see above) and digested with Thermolysin (Promega) (1:5 w/w), in 50 mM Tris-HCl, 0.5 mM CaCl_2_ (75°C, overnight).

### Phosphopeptide enrichment

Samples were suspended in loading buffer [50% acetonitrile (ACN), 0.1% trifluoroacetic acid (TFA)], incubated with PHOS-Select iron affinity beads (Sigma Aldrich) at a ratio 1/2.5 (beads volume μL/peptide mixture μg) for 30 min and loaded into a 200 μL Gel-Loader tip (Costar). After washing with the binding and washing (20% ACN, 1% TFA) buffers, phosphopeptides were eluted with 4% ammonium hydroxide, desalted on Graphite spin-column (Pierce-Thermo Fisher Scientific) and dried (Speed-vacuum).

### LC-MS/MS analysis

Samples were analyzed using an Ultimate 3000 nano-RSLC (Thermo Scientific, San Jose California) coupled in line with an LTQ-Orbitrap ELITE mass spectrometer *via* a nano-electrospray ionization source (Thermo Scientific, San Jose California).

Peptides were loaded on a C18 Acclaim PepMap100 trap-column (75 μm ID x 2 cm, 3 μm, 100Å, Thermo Fisher Scientific) for 3.5 minutes at 5 μL/min with 2% ACN, 0.1% formic acid (FA) in H_2_O and then separated on a C18 Accucore nano-column (75 μm ID x 50 cm, 2.6 μm, 150Å, Thermo Fisher Scientific) with a linear gradient from 5% to 50% buffer B (A: 0.1% FA in H_2_O / B: 80% ACN, 0.08% FA in H_2_O). For nuclear and cytosolic extracts analyses the gradient duration was 480 minutes before phospho-enrichment and 240 minutes after phosphoenrichment. For RARα IP analysis, gradient duration was 480 minutes, before and after phospho-enrichment, because thermolysin digestion produces a higher number of peptides than trypsin digestion. The total duration was set to 520 (or 280) minutes at a flow rate of 200 nL/min and at 40°C.

The mass spectrometer was operated in data-dependent mode with survey scans from m/z 300–1600 acquired in the Orbitrap at a resolution of 120,000 at m/z 400. The 15 most intense peaks from survey scans were selected for further fragmentation in the LTQ with an isolation window of 2.0 Da and were fragmented by CID with a normalized collision energy of 35%. Unassigned and single charged states were rejected. To enhance phosphorylation detection, Multi-Stage Activation was enabled and the corresponding neutral loss masses were 32.66; 49.00; 65.33 and 98.00.

The Ion Target Value for the survey scans in the Orbitrap and the MS2 mode in the LTQ were set to 1E6 and 5E4 respectively and the maximum injection time was set to 100 ms for both scan modes. Dynamic exclusion was used. Exclusion duration was set to 30 s, repeat count was set to 1 and exclusion mass width was ± 10 ppm.

Proteins were identified by database searching using SequestHT with Proteome Discoverer 1.4 software (Thermo Fisher Scientific) against the Human Swissprot database (2013–06 release, 20218 entries). Precursor and fragment mass tolerance were set at 7 ppm and 0.5 Da respectively, and up to 2 missed cleavages were allowed. Oxidation (M) and Phosphorylation (S, T, and Y) were set as variable modification, and Carbamidomethylation (C) as fixed modification.

Peptides from nuclear and cytosolic extracts were filtered with a 1% FDR (false discovery rate) using the “Target Decoy” Proteome Discoverer’s node and rank 1. Trypsin was defined to cut in C-terminus of Lysine (K) and Arginine (R), whereas Chymotrypsin was defined to cut in C-terminus of Phenylalanine (F), Tryptophan (W), Tyrosine (Y) and Leucine (L). Phosphorylation sites were validated using the PhosphoRS 3.0 node, with at least 99% phospho-site probability. Exported phosphopeptides were then processed using ProteinModificationToolkit, with a cutoff value at 99%, and automatically sent to Motif-x with the following parameters: Occurrences 20 / Significance P < 10^−6^ / Background IPI Human Proteome.

Peptides from RARα immunoprecipitation were filtered with a score versus charge state (1.5 z_1_, 2.5 z_2_, 3 z_3_ and 3.2 z_≥4_, Proteome Discoverer’s recommendations) because dataset was not sufficient to apply FDR and rank 1. Thermolysin was defined to cut in C-terminus of Valine (V), Alanine (A), Methionine (M), Isoleucine (I), Leucine (L) and Phenylalanine (F). RARα phosphopeptides spectra were manually inspected and RARα phosphorylation sites were manually validated.

### High throughput mRNA sequencing (RNA-seq)

Total RNA was extracted from MCF7 and BT474 cells treated or not with RA for 4h. A library of template molecules suitable for high throughput DNA sequencing was created following the Illumina “Truseq RNA sample preparation low throughput” protocol with some modifications, as previously described [24]. Briefly, mRNA was purified from 4 μg total RNA using oligo-dT magnetic beads and fragmented using divalent cations at 94°C for 8 minutes. The cleaved mRNA fragments were reverse transcribed to cDNA using random primers, and then the second strand of the cDNA was synthesized using Polymerase I and RNase H. The next steps of RNA-Seq Library preparation were performed in a fully automated system using SPRIworks Fragment Library System I kit (ref A84801, Beckman Coulter, Inc.) with the SPRI-TE instrument (Beckman Coulter, Inc.). Briefly, in this system double stranded cDNA fragments were blunted, phosphorylated and ligated to indexed adapter dimers, and fragments in the range of ~200–400 bp were size selected. The automated steps were followed by PCR amplification (30 sec at 98°C; [10 sec at 98°C, 30 sec at 60°C, 30 sec at 72°C] x 12 cycles; 5 min at 72°C), and then surplus PCR primers were removed by purification using AMPure XP beads (Agencourt Biosciences Corporation). DNA libraries were checked for quality and quantified using 2100 Bioanalyzer (Agilent). The libraries were loaded in the flow cell at 7pM concentration and clusters generated and sequenced in the Illumina Genome Analyzer IIX as single-end 54 base reads.

Image analysis and base calling were performed using CASAVA v1.7.0. Sequence Reads were mapped onto the hg19 assembly of the human genome by using Tophat v1.4.1 [25] and the bowtie v0.12.7 aligner. Gene expression was quantified using Cufflinks v1.0.1 [26] and gene annotations from Ensembl release 62. Normalization and statistical analysis were performed with the method proposed by Anders and Huber [27] and implemented in the DESeq Bioconductor package. P-values were adjusted for multiple testing using the Benjamini and Hochberg [28] method. Only genes with |log2 fold-change| > 1 and adjusted p-value < 0.05 were considered. Functional analyses of these genes were performed using the Manteia program [29].

### Motif research

The gene regions located ±10 kb from the gene limits (Ensembl release 62) were analyzed using regular expression searches to detect perfect consensus 5’-RGKTSA-3’ half sites with the different spacings. The potential RAR binding elements were aligned on the same strand to ensure the sense and antisense matches gave homogeneous positions.

### Chromatin Immunoprecipitation (ChIP) experiments

They were performed as described previously [20]. Primer sequences are available upon request.

### Cell proliferation, immunoprecipitation and immunoblotting

Cell proliferation was analyzed by using the XTT (2,3bis-(2methoxy-4-nitro-5sulfophenyl)-2H-tetrazolium-5-carboxanilide) assay kit (Roche Diagnostic).

Immunoprecipitation and immunoblotting were described previously [30]. Rabbit polyclonal antibodies against RARα, RPα(F) were described earlier [23]. Mouse monoclonal antibodies recognizing specifically RARα phosphorylated at positions S74 and S77 (Ab36α) were generated by immunization of BALB/c mice with synthetic phosphopeptides coupled to ovalbumin according to standard procedures. Specificity was checked by ELISA with synthetic peptides either non phosphorylated or phosphorylated at S77, S74 or at both S74 and S77.

Mouse embryo fibroblasts (MEFs) expressing RARα WT or RARαS77A in a RAR(α, β, γ) null background were previously described [21].

## Results

### Strategy to compare the phosphorylated proteins in MCF7 and BT474 cells

**Fig 1:**
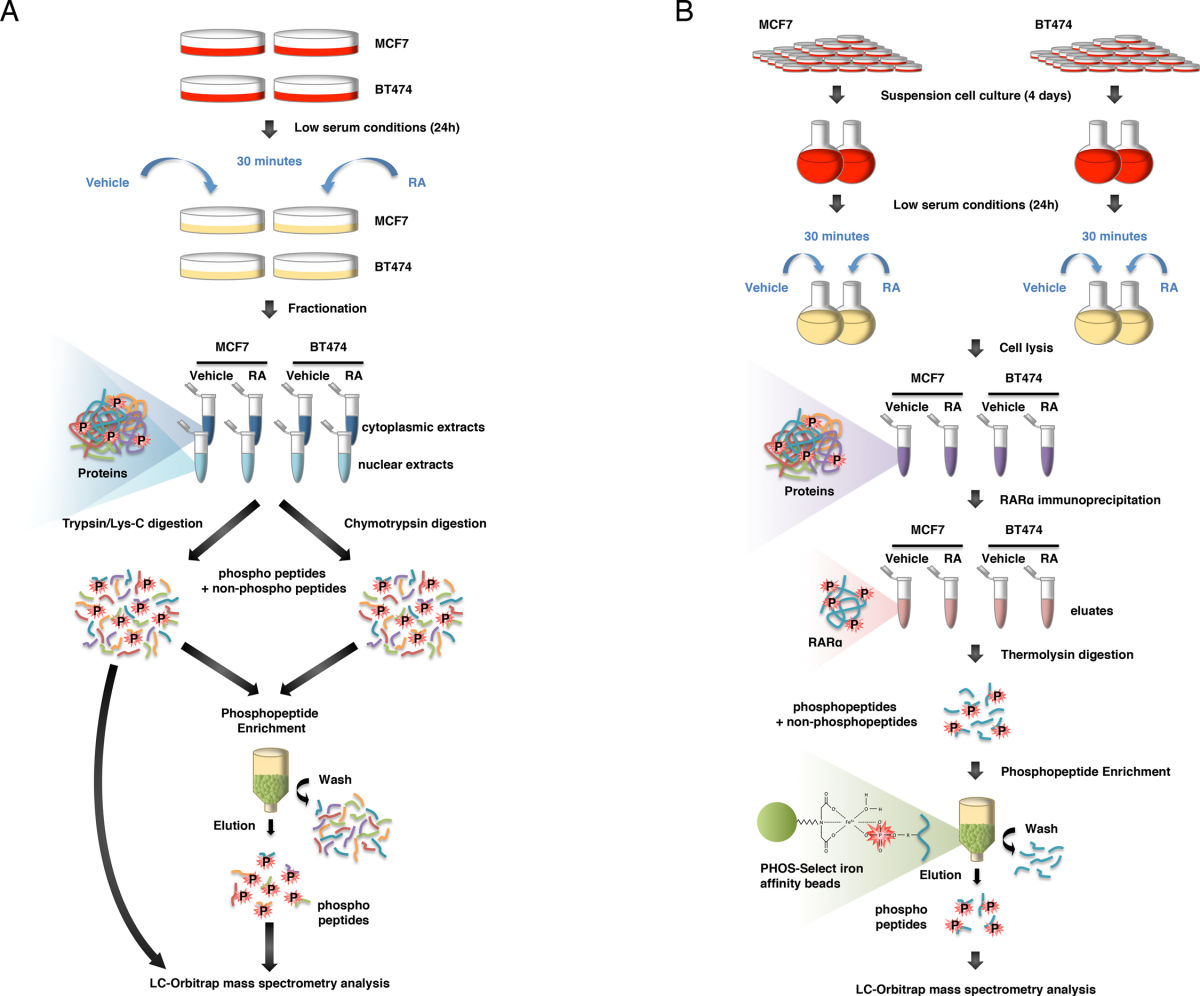
Workflow for the phosphoproteomics strategy. (A) Phosphoproteome. Nuclear and cytoplasmic extracts were prepared and divided in two: one half was digested with trypsin/Lys-C and the other half with chymotrypsin. A small fraction of the trypsin/Lys-C digests was analyzed directly without further purification. The remaining digests were subjected to phosphopeptide enrichment and MS analysis. (B) RARα phosphorylation. Whole cell extracts were prepared from MCF7 and BT474 cells with and without a 30 min RA treatment. RARα was immunoprecipitated and the eluates were thermolysin-digested. Phosphopeptides were enriched and analyzed by nano-LC-LTQ-Orbitrap MS.

In order to analyze whether the phosphoproteome is affected in the RA-resistant BT474 cells, compared to the RA responsive MCF7 cells, we performed a large-scale nano-LC-LTQ-Orbitrap MS approach (Fig 1A). As the effects of RA on the signaling pathways are very rapid [9, 21], the cells were treated with RA or the solvent for a short time (30 min).

Nuclear and cytosolic extracts were prepared from both cell lines, treated or not with RA, in two replicate experiments. Extracts purity was checked by immunoblotting of β-tubulin and lamin A/C which are present exclusively in the cytosol and in the nucleus respectively (S1 Fig). Purity was also checked with GO statistics using the Manteia program [29]. Extracts were divided in two for different proteolytic digestions. One half was digested with trypsin in combination with Lys-C, which both cleave peptides carboxyterminal of the amino acids Lys and Arg. Such a combination reduces missed cleavages due to inhibition of trypsin under the denaturing conditions required for digestion of tightly folded proteins. The other half was digested with chymotrypsin, which cleaves peptides carboxy terminal of aromatic (Tyr, Trp, Phe) or hydrophobic (Leu) amino acids. Combined data obtained from two different proteolytic digests with different cleavage patterns increase sequence coverage [31]. This is important especially when localization of a modification is desired rather than simple protein identification.

Then an aliquot of the trypsin/Lys-C digested samples was submitted directly, without phosphopeptide enrichment, to nano-LC-LTQ-Orbitrap MS, in order to analyze the overall protein composition of the extracts. For each replicate experiment, only the proteins identified with at least two peptides with high confidence in both the vehicle- and RA- treated extracts were selected (S1 and S2 Tables). Then the remaining trypsin/Lys-C-digested samples and the chymotrypsin-digested ones were submitted to phosphopeptide enrichment and analyzed. The results of the two digestion protocols were combined and the phosphopeptides corresponding to the proteins selected above without phosphoenrichment were considered. Proteins with at least one phosphorylated peptide with high confidence were considered (S1 and S2 Tables).

### Phosphopeptides analysis

**Fig 2:**
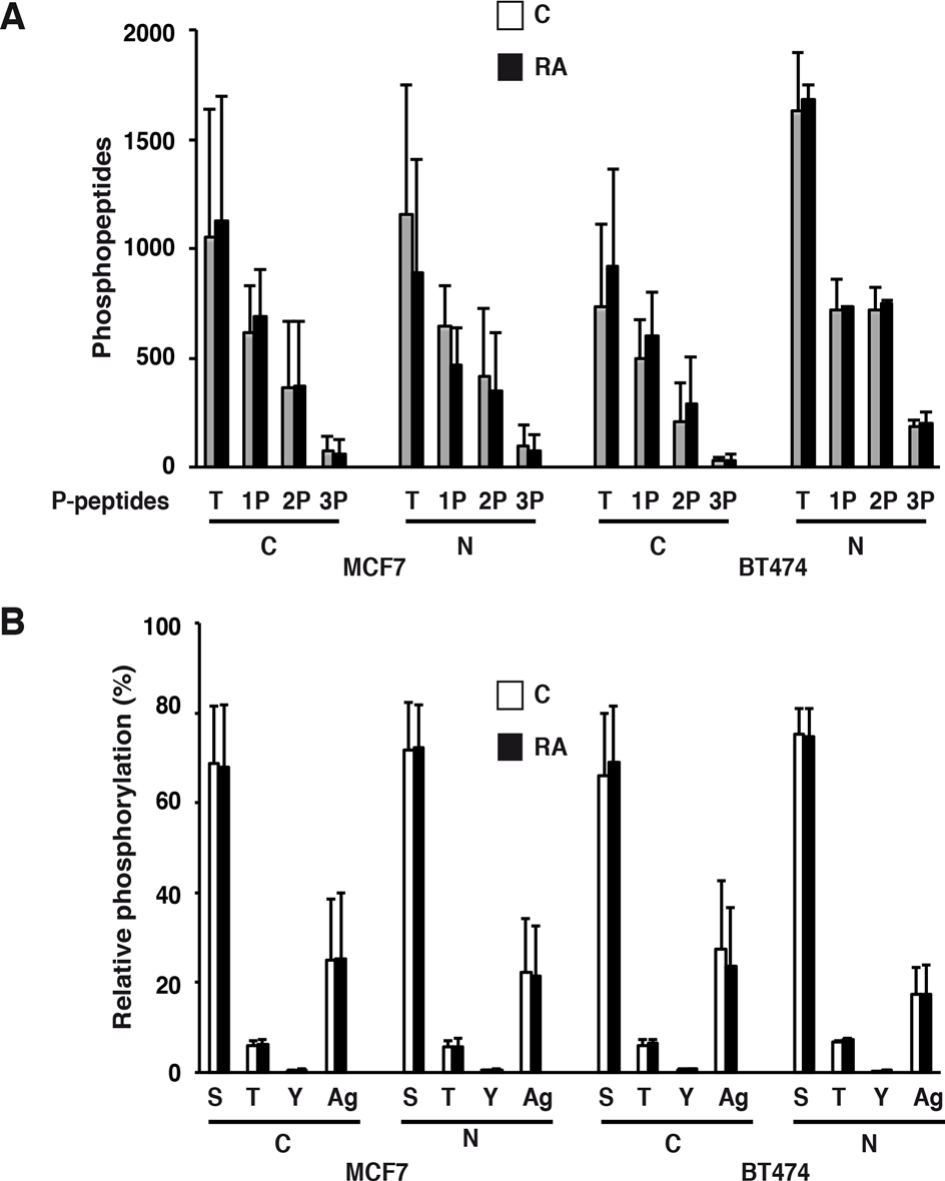
Overview of phosphorylation in MCF7 and BT474 cells. (A) Relative frequency of mono- (1P), bi-(2P) and tri- (3P) phosphorylated peptides in the cytosolic and nuclear extracts of MCF7 and BT474 cells with and without RA treatment. T: total number of phosphopeptides. C: cytosolic extracts, N: nuclear extracts. (B) Relative phosphorylation of Serine (S), Thr (T) and Tyr (Y) residues (Ag: ambiguous). The values are the average ±SD of two experiments.

Patterns among the identified unique phosphopeptides were examined (see materials and methods) and were found to be very similar in both cell lines and in the two experiments (S3 and S4 Tables). First, the number of phosphorylated residues within each phosphopeptide was analyzed. The majority of the phosphopeptides contained 1 or 2 sites (Fig 2A). Only 5% contained 3 phosphorylated sites. Moreover, similar to other studies [32, 33], we observed mostly Ser phosphorylation (70%), followed by Thr (5%) and Tyr (0,4%) (Fig 2B). In both cell lines, RA treatment affected neither the preference for Ser phosphorylation nor the percentage of mono- and bi-phosphorylated peptides (Fig 2A and 2B). The motifs of the phosphopeptides were also examined and proline-directed (SP) and acidic sites (SD and SE) were found to be the most common (S2 Fig). Then we examined the number of phosphorylated sites within each phosphoprotein. In both cell lines, we observed that 50% of the phosphoproteins are phosphorylated at 1 or 2 residues (S3 Fig). Only a few proteins (1%) exemplified by the serine/arginine repetitive matrix proteins 1 and 2 (SRRM1 and SRRM2) carried more than 10 sites.

### Comparison of the cytosolic phosphoproteome

**Fig 3:**
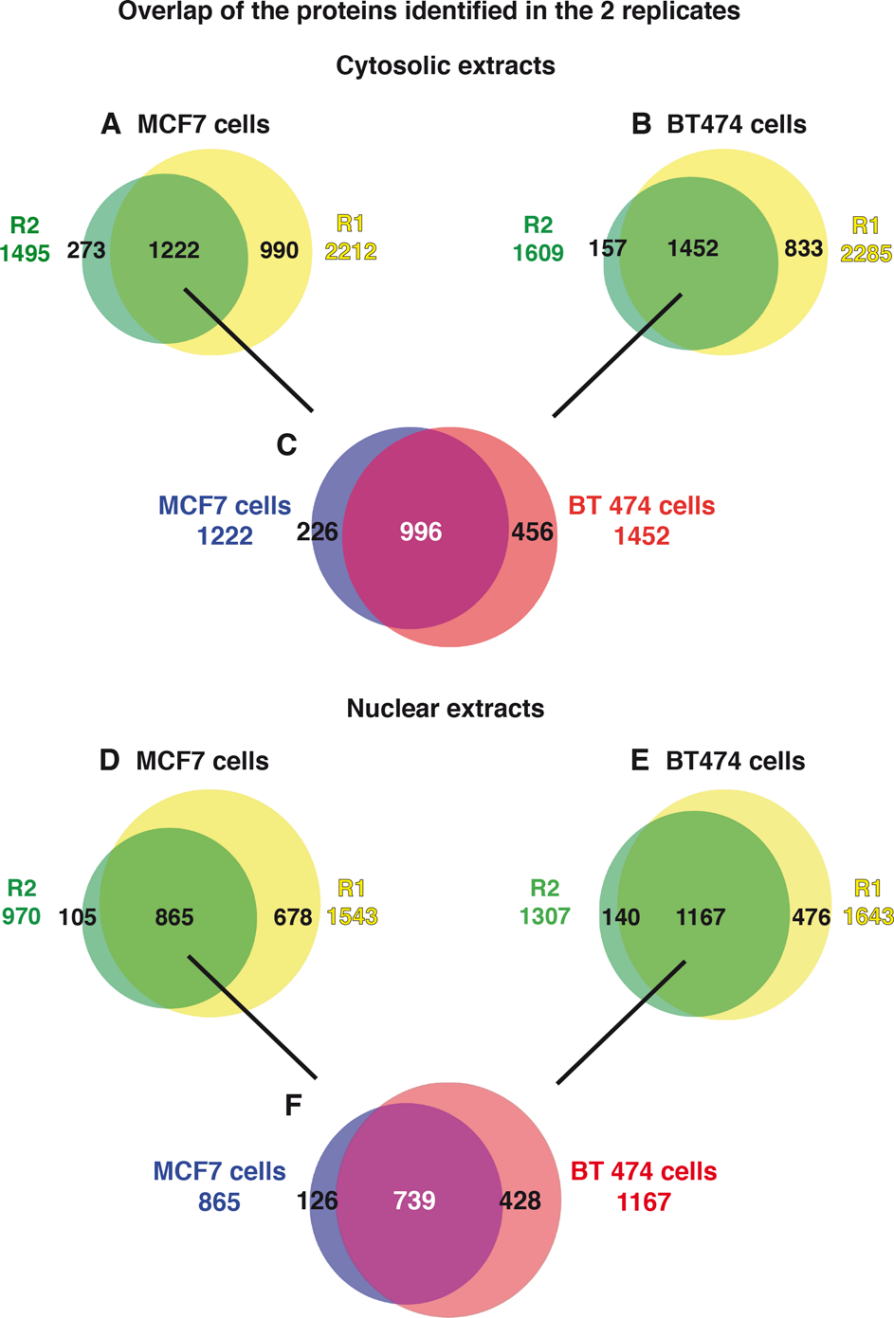
Overlap of the total proteins identified in the two replicate experiments R1 and R2. (A) Overlap of the MCF7 cytosolic proteins identified in the replicate experiments R1 and R2 (B) Overlap of the BT474 cytosolic proteins identified in R1 and R2 (C) Overlap of the MCF7 and BT474 cytosolic proteins. (D) Overlap of the MCF7 nuclear proteins identified in R1 and R2 (E) Overlap of the BT474 nuclear proteins identified in R1 and R2 (C) Overlap of the MCF7 and BT474 nuclear proteins.

**Fig 4:**
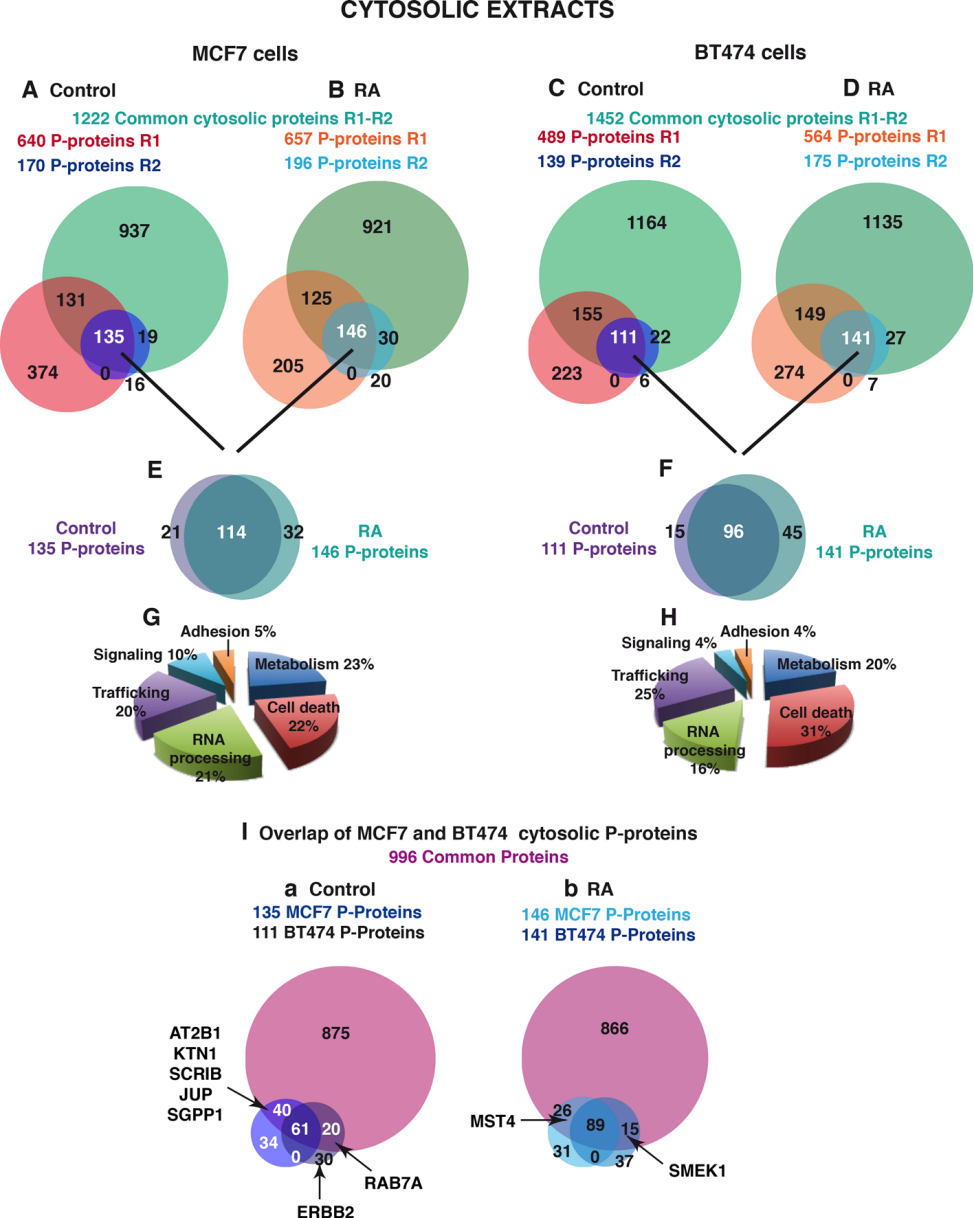
Comparison of the phosphorylated proteins in the cytosolic extracts of MCF7 and BT474 cells. (A and B) Overlap of the cytosolic phosphorylated proteins of MCF7 cells identified in the two replicate experiments R1 and R2. The phosphoproteins identified in the cytosolic extracts of MCF7 cells without (A) or with (B) RA treatment, in each replicate, were crossed with each other and with the common proteins identified in Fig 3. (C and D) Same for BT474 cells. (E and F) Overlap of the phosphorylated proteins identified in the absence and in the presence of RA in the cytosolic extracts of MCF7 (E) and BT474 cells (F). (G and H) 3D pie charts showing the biologic functions of the cytosolic P-proteins identified in MCF7 (G) and BT474 (H) cells. (I) Overlap of the cytosolic phosphoproteins identified in MCF7 and BT474 cells in the absence (a) and in the presence of RA (b).

For each cell line, we selected the proteins identified in both the vehicle- and RA- treated cytosolic extracts and in the two replicate experiments. Using this strategy, 1222 and 1452 proteins were identified in the cytosolic extracts of MCF7 and BT474 cells respectively in both replicates (Fig 3A and 3B). Then, for each cell line, the phosphorylated proteins detected in each replicate experiments were crossed not only with each other but also with the total proteins detected in the two replicates (Fig 4A and 4B).

In the controls, 135 phosphoproteins were scored in MCF7 cells (Fig 4A) and 111 in BT474 cells (Fig 4C), in both replicates. This indicates that around 11% of the MCF7 cytosolic proteins and 8% of the BT474 ones are phosphorylated. These phosphorylated cytosolic proteins were assigned to functional groups, according to enriched Gene Ontology (GO), using the Manteia program (http://manteia.igbmc.fr) [29]. This analysis identified in both cell lines, proteins involved in metabolism, cell death, RNA processing, trafficking/transport, signal transduction and cell adhesion (Fig 4G and 4H).

Next, the same strategy was followed to analyze the proteins phosphorylated after RA treatment. In both cell types, RA treatment affected only mildly the % of cytosolic phosphoproteins (Fig 4B and 4D). Indeed, 80% of the phosphoproteins identified with and without RA treatment overlapped (Fig 4E and 4F).

Then, the cytosolic phosphoproteins profiles of the two cell lines were compared. First, the proteins detected in both cell lines were selected (Fig 3C). Then the MCF7 and BT474 phosphoproteins selected above were crossed not only with each other but also with the overall proteins common to both cell lines (Fig 4I).

**Fig 5:**
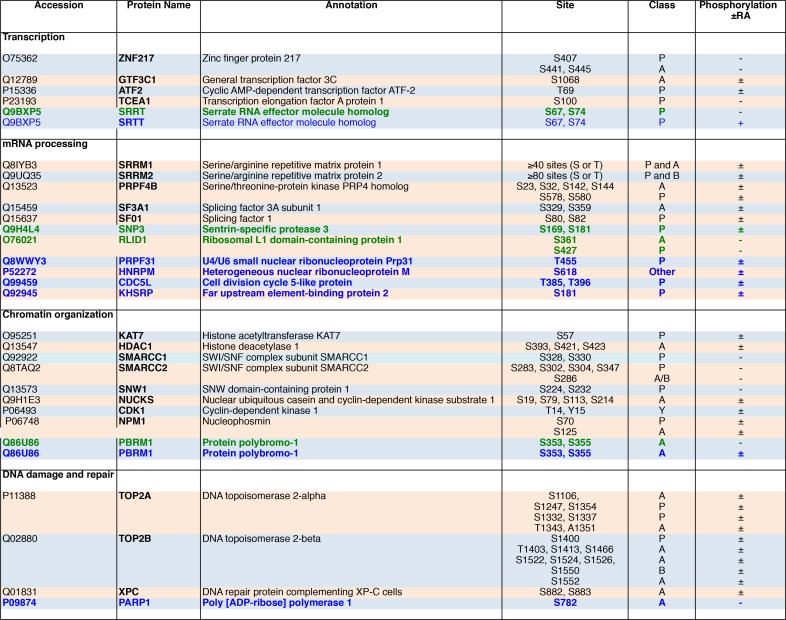
List of phosphoproteins, grouped per biological functions that were detected in the cytosolic extracts of MCF7 and/or BT474 cells, in the two replicate experiments. For each protein, the phosphopeptides were analyzed manually. The site classes are assigned as acidic (A), basic (B) or proline-directed (P). Phosphoproteins detected in both cell lines (Black), in MCF7 cells only (Green) or in BT474 cells only (Blue). Indicated is whether phosphorylation occurs in the absence of RA only (-), in the presence of RA only (+) or both in the absence and presence of RA (±).

In the absence of RA, 61 proteins (corresponding to around 50% of the phosphoproteins identified in each cell line) were phosphorylated in both MCF7 and BT474 cells in both replicates (Fig 4Ia and Fig 5). Note that several proteins involved in Trafficking (AT2B1, KTN1), adhesion/cell junctions (SCRIB, JUP), or in metabolism (SGPP1), were phosphorylated in MCF7 cells only in both replicates. Others such as the Ras-related GTP binding protein Rab-7a were phosphorylated in BT474 cells only (Fig 4Ia and Fig 5).

RA treatment increased by 30% the number of proteins phosphorylated in both cell lines (Fig 4Ib). Interestingly, in MCF7 only, RA induced the phosphorylation of MST4 (Fig 4Ib and Fig 5), which is involved in the activation of MAPKs [34, 35], corroborating that RA activates this pathway [9, 21]. In contrast, in BT474 cells only, RA induced the phosphorylation of SMEK1, a subunit of the Ser/Thr protein phosphatase 4, which interferes with the PI3K/Akt pathway [36]. In BT474 cells only, RA also inhibited the phosphorylation of RAB7A (Fig 4Ib). Altogether these results indicate that the cytosolic phosphoproteome of MCF7 cells differs from that of BT474 cells. They also indicate that RA differentially impacts the cytosolic phosphoproteome of both cell lines. Interestingly, around 30 phosphoproteins were detected in one cell line but not in the other one and reciprocally. The best example is the erb-b2 receptor tyrosine kinase that was overexpressed and phosphorylated in BT474 cells only and was not detected in MCF7 cells (Fig 4Ia and Fig 5).

### Comparison of the nuclear phosphoproteome

The same strategy was followed for the nuclear extracts and 865 and 1167 proteins were scored in MCF7 and BT474 cells respectively, in both experiments (Fig 3D and 3E).

**Fig 6:**
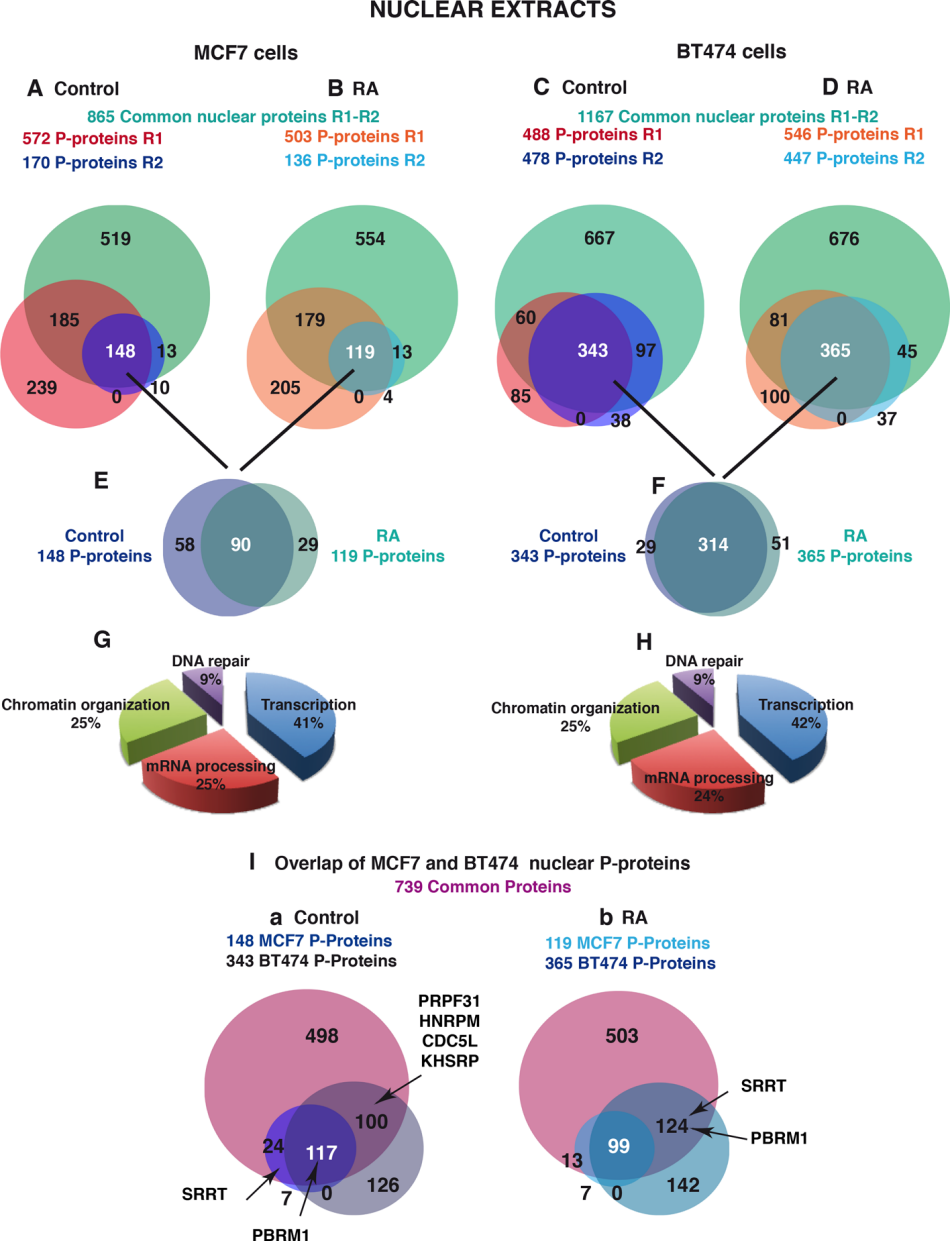
Comparison of the phosphorylated proteins in the nuclear extracts of MCF7 and BT474 cells. (A and B) Overlap of the phosphorylated nuclear proteins of MCF7 cells identified in the two replicate experiments R1 and R2, in the absence (A) or in the presence (B) of RA. (C and D) Same for the phosphoproteins identified in the nuclear extracts of BT474 cells. (E and F) Overlap of the nuclear phosphorylated proteins identified in the absence and in the presence of RA in MCF7 (E) and BT474 cells (F). (G and H) 3D pie charts showing the biological functions of the nuclear phosphoproteins identified in MCF7 (G) and BT474 (H) cells. (I) Overlap of the nuclear phosphoproteins identified in MCF7 and BT474 cells in the absence (a) and in the presence of RA (b).

In the untreated MCF7 cells, 148 phosphoproteins were scored in the two experiments (Fig 6A), corresponding to around 17% of the overall proteins. In BT474 cells, 343 phosphorylated proteins were scored (Fig 6C), representing a higher percentage (29%) than in MCF7 cells. In both cell types, RA treatment affected only mildly the % of phosphoproteins (Fig 6B, 6D, 6E and 6F). According to enriched Gene Ontology, these nuclear phosphorylated proteins are involved in transcription DNA-templated, chromatin modifications/organization, mRNA processing (splicing) and DNA damage and repair (Fig 6G and 6H).

**Fig 7:**
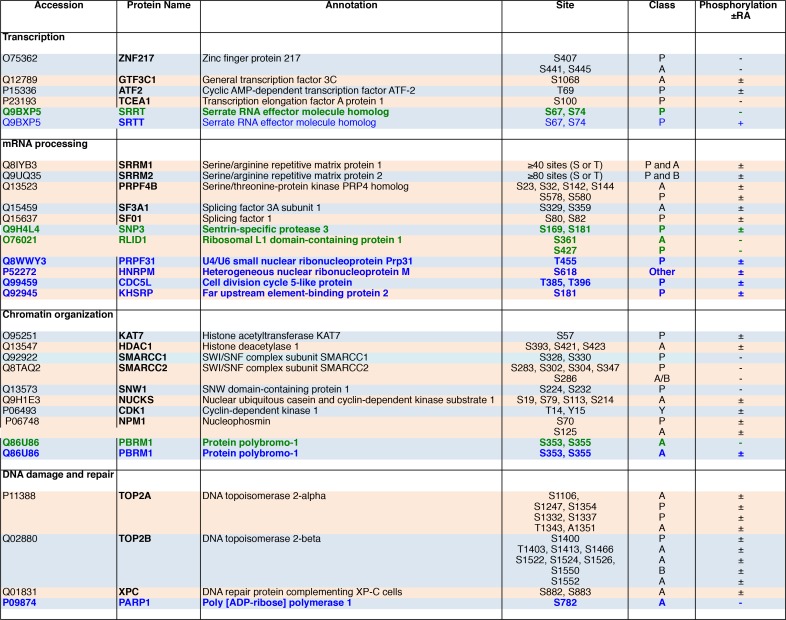
List of phosphoproteins, grouped per biological functions that were detected in the nuclear extracts of MCF7 and/or BT474 cells, in the two replicate experiments. For each protein, the phosphopeptides were analyzed manually. The site classes are assigned as acidic (A), basic (B) or proline-directed (P). Phosphoproteins detected in both cell lines (Black), in MCF7 cells only (Green) or in BT474 cells only (Blue). Indicated is whether phosphorylation occurs in the absence of RA only (-), in the presence of RA only (+) or both in the absence and presence of RA (±).

We next compared the two cell lines (Fig 3E, Fig 6I and Fig 7). The number of nuclear proteins phosphorylated in both cell lines represented 80% of the MCF7 phosphoproteins but only 34% of the BT474 ones (Fig 6Ia). Consequently several proteins involved in mRNA processing (PRPF31, HNRPM, CDC5L and KHSRP) or in DNA repair (PARP1) were phosphorylated in BT474 cells only. The other interesting point is that depending on the cell type, a same protein responded differently to RA (Fig 6Ia and 6Ib and Fig 7). As an example, the protein polybromo-1 (PBRM1) was phosphorylated in both cell lines in the absence of RA but in BT474 only after RA addition. In contrast the Serrate RNA effector molecule homolog (SRRT) was phosphorylated in MCF7 cells only in the absence of RA and in BT474 cells only in the presence of RA. Altogether, these results indicate that RA also differentially impacts the nuclear phosphoproteome of MCF7 and BT474 cells.

### RARα phosphorylation analysis

Both MCF7 and BT474 cells express RARα. However the RARα protein, phosphorylated or not, could not be detected in the above nano-LC-LTQ-Orbitrap MS approach, indicating that its abundance was too low for detection under these conditions and/or that digestion was not efficient. Therefore, we performed a large-scale culture of the cells and RARα was enriched by immunoprecipitation (see material and methods) before proteolytic digestion and nano-LC-LTQ-Orbitrap MS analysis (Fig 1B). Two independent biological replicates were performed for each cell line with and without RA treatment (30 min). These conditions allowed the detection of the RARα protein without phosphoenrichment.

**Fig 8:**
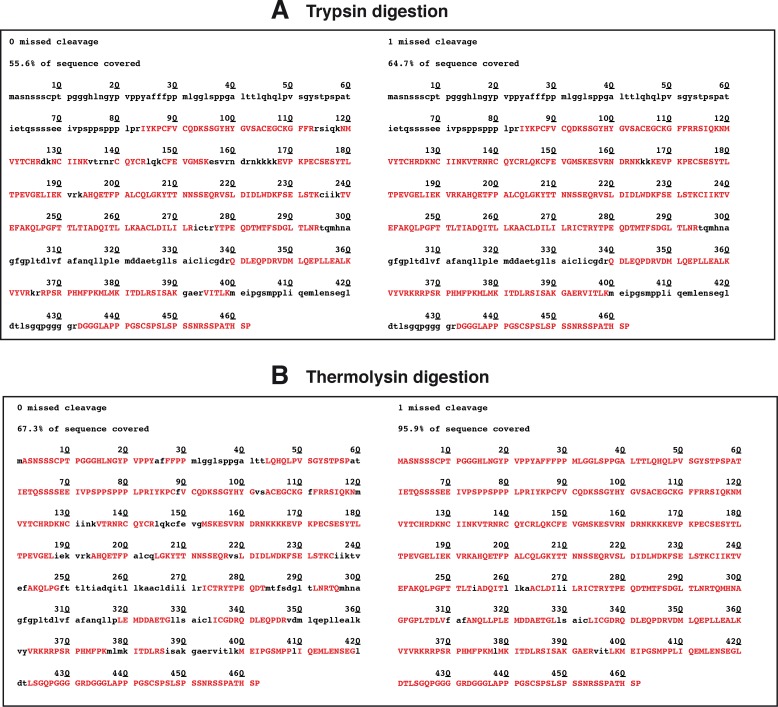
**RARα sequence coverage upon digestion with trypsin (A) or thermolysin (B).** Simulation was performed using PeptideMass Peptide characterization software (Expasy). In red are the obtained peptides.

Interestingly, digestion simulations indicated that trypsin digestion was not convenient for the detection of RARα phosphopeptides. Indeed, trypsin generated multiple peptides outside of the detectable size range and the N-terminal domain (NTD) was not covered (Fig 8). Consequently, after phosphopeptide enrichment, no or very few RARα phosphopeptides were detected, suggesting that the potential phosphorylation sites were mainly located in the non-covered sequence. Therefore we searched for better enzymes and we selected thermolysin, which cleaves at the N-terminus of the hydrophobic residues Leu, Phe, Val, Ile, Ala and Met. This enzyme led to higher sequence coverage, especially in the NTD of RARα, and generated properly sized peptides embedding the phosphorylation sites (Fig 8). Finally, the phosphopeptides obtained after thermolysin digestion were enriched on PHOS-Select beads and analyzed by nano-LC-LTQ-Orbitrap MS (S5 Table).

**Fig 9:**
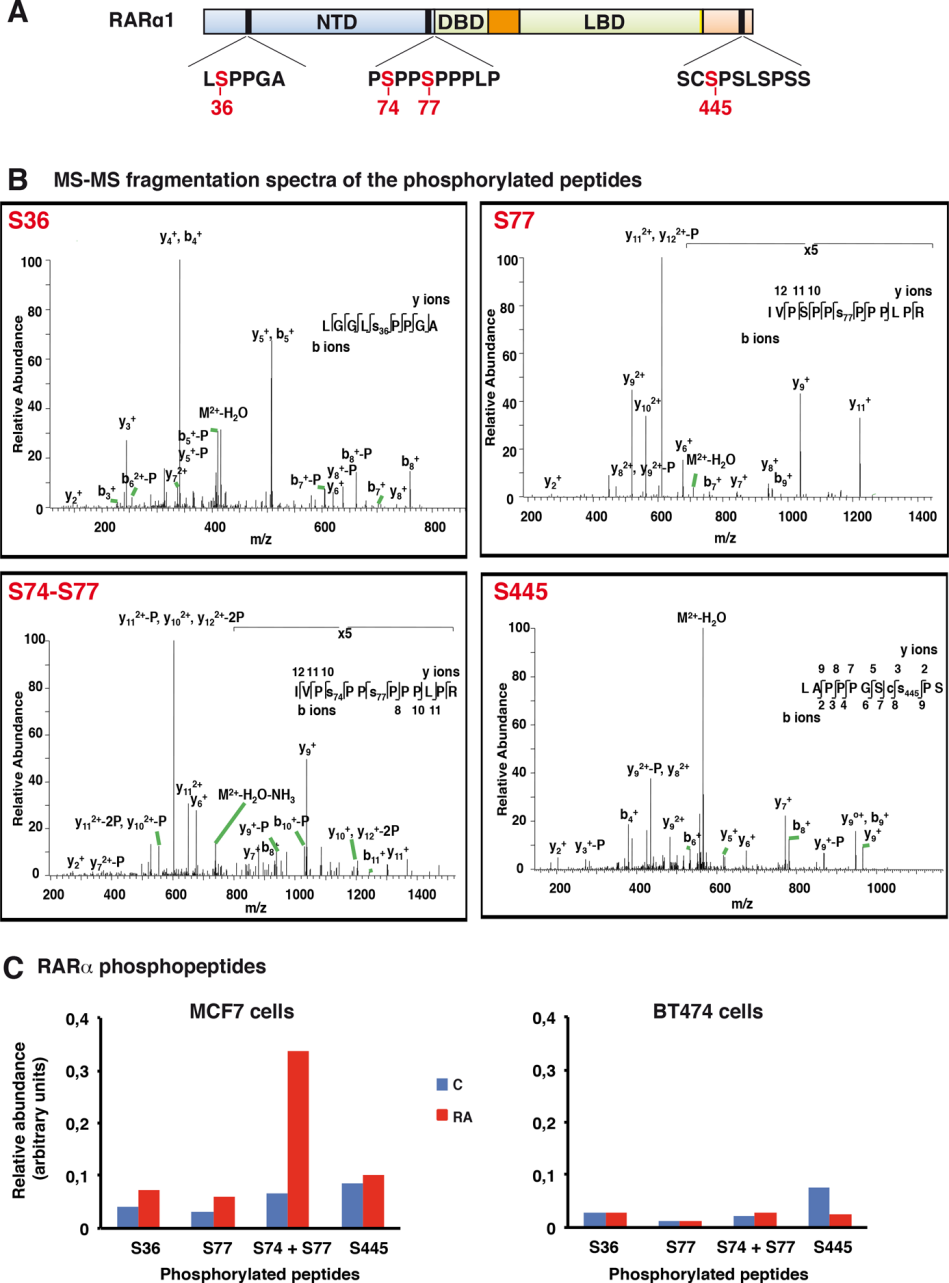
Comparison of the RARα phosphorylation sites in MCF7 and BT474 cells. (A) Schematic representation of the RARα domains with the S36, S74, S77 and S445 phosphosites. (B) MS-MS fragmentation spectra of the phosphorylated peptides. (C) Relative abundance of the RARα phosphopeptides in MCF7 (left) and BT474 cells (right) with and without RA treatment. Peptides numbers were normalized to the protein content of the immunoprecipitation eluates. The values are the average ±20% of two separate experiments.

In the two cell lines, in the absence of RA, we identified peptides mono-phosphorylated at Ser residues located in the NTD (S36, S77) and in the C-terminal domain (S445) (Fig 9A and S5 Table). Peptides bi-phosphorylated at S77 and S74 were also identified. Note that no peptides monophosphorylated at S74 were detected. The MS-MS spectra of the phosphorylated peptides are shown in Fig 7B. S77 and S445 phosphorylation was already found in a previous study [37]. However, S36 is a novel N-terminal proline-directed phosphorylation site. The other novelty is the detection of peptides biphosphorylated at S74 and S77.

**Fig 10:**
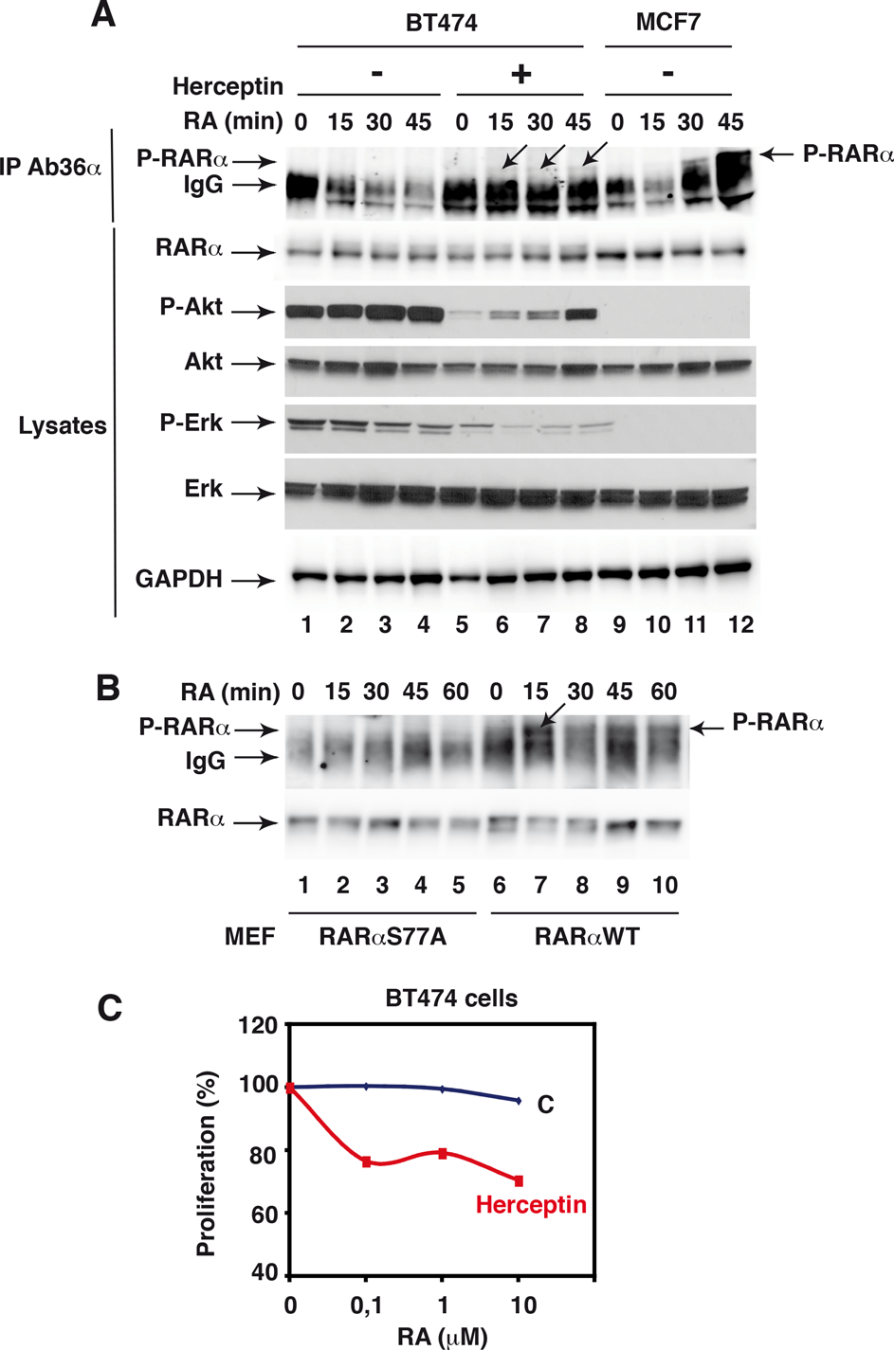
Validation of RARα phosphorylation at S74 and S77. (A). Kinetics experiments showing that RA induces the phosphorylation of RARα at both S74 and S77 in MCF7 cells and not in BT474 cells and that Herceptin (100μg/ml) restores partially RARα phosphorylation. Cell extracts were immunoprecipitated with antibodies recognizing specifically RARα phosphorylated at both S74 and S77 (Ab36α). The eluates were resolved by SDS-PAGE and immunoblotted with RPα(F). Herceptin also decreases the phosphorylation of Akt and Erks. An arrow indicates the band corresponding to the phosphorylated form of RARα. (B). Kinetics experiments showing that RA also induces the phosphorylation of RARα at both S74 and S77 in MEFs expressing RARαWT in a triple RAR null background. No increase was observed in MEFs expressing RARαS77A. Extracts were immunoprecipitated with Ab36α as in A. (C) Herceptin restores the antiproliferative effect of RA on BT474 cells. When 50% confluent, BT474 cells were treated with increasing concentrations of RA in the absence or presence of Herceptin (100μg/ml). Cell proliferation was analyzed after 48h using the XTT (2,3bis-(2methoxy-4-nitro-5sulfophenyl)-2H-tetrazolium-5-carboxanilide) assay kit.

After RA addition, the number of peptides phosphorylated at S36 and S445 did not change significantly in both cell lines (Fig 9C). The number of peptides monophosphorylated at S77 did not change either. Remarkably, in MCF7 cells, the number of peptides bi-phosphorylated at S74 and S77 increased markedly (Fig 9C). That RA increases the phosphorylation of both S74 and S77 in MCF7 cells was corroborated in immunoprecipitation experiments performed with monoclonal antibodies recognizing specifically RARα phosphorylated at both residues (Fig 10A, lanes 9–12).

Such results were unexpected since we previously reported only an increase in S77 phosphorylation in MCF7 cells as well as in mouse embryonic fibroblasts (MEFs) [21]. Therefore we performed additional studies taking advantage of MEFs expressing RARαWT or RARα with S77 substituted with an alanine (RARαS77A) in a triple RAR null background [21]. In immunoprecipitation experiments performed with our phosphor antibodies, we found that RA induced the phosphorylation of RARα at both S74 and S77 in MEF expressing RARα WT (Fig 10B, lanes 6–10). However, no signal was obtained with MEFs expressing RARαS77A (Fig 10B, lanes 1–5). Such results suggest that phosphorylation of S74 would depend on that of S77 and explain why in our previous studies combining mutagenesis and phosphopeptide mapping the substitution of S77 with an alanine induced the disappearance of the phosphorylated peptide while the substitution of S74 did not [37].

In contrast, in BT474 cells, the number of peptides bi-phosphorylated at S74 and S77 did not increase upon RA addition (Figs 9C and 8A lanes 1–4). Most interestingly, a pretreatment of BT474 cells with Herceptin, which restores the antiproliferative effect of RA (Fig 10C) and reverses the phosphorylation of Akt and Erks downstream of erbB-2 (Fig 10A), also restored partially the RA-induced phosphorylation of RARα (Fig 10A, lanes 6–8). Such results indicate that the absence of effect of RA on RARα phosphorylation would reflect at least in part ERB-B2 overexpression.

### Genome-wide comparison of the RA-regulated genes

**Fig 11:**
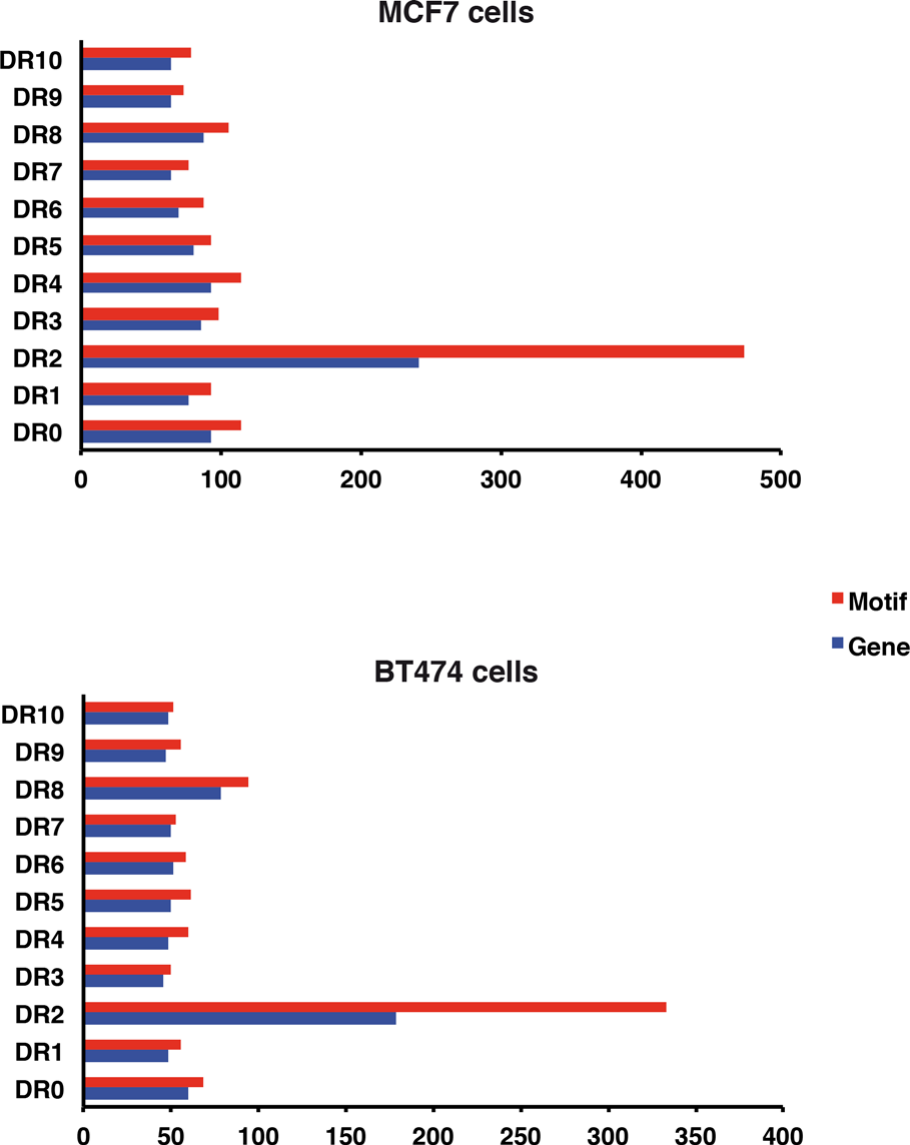
DR (DR0-DR10) in the MCF7 and BT474 RA-regulated genes. The frequencies of the different DRs and of genes with DRs are shown.

In previous studies, we reported that the phosphorylation of RARα and several other factors is required for the RA-induced activation of canonical RA targets genes [21, 22, 37]. From the above results one can hypothesize that the regulation of the RA target genes would be different in MCF7 and BT474 cells. Therefore the MCF7 and BT474 cells were compared for their repertoire of RA-regulated genes (RA-treated versus untreated), in high throughput sequencing experiments. For each cell line, a list of up- and down-regulated genes was generated (S6 Table). The analysis of the regions located ±10 kb from the transcription start sites revealed the presence of direct repeats (DRs) spaced by 0 to 10 base pairs, corroborating that these genes are RA-regulated genes (Fig 11).

**Fig 12:**
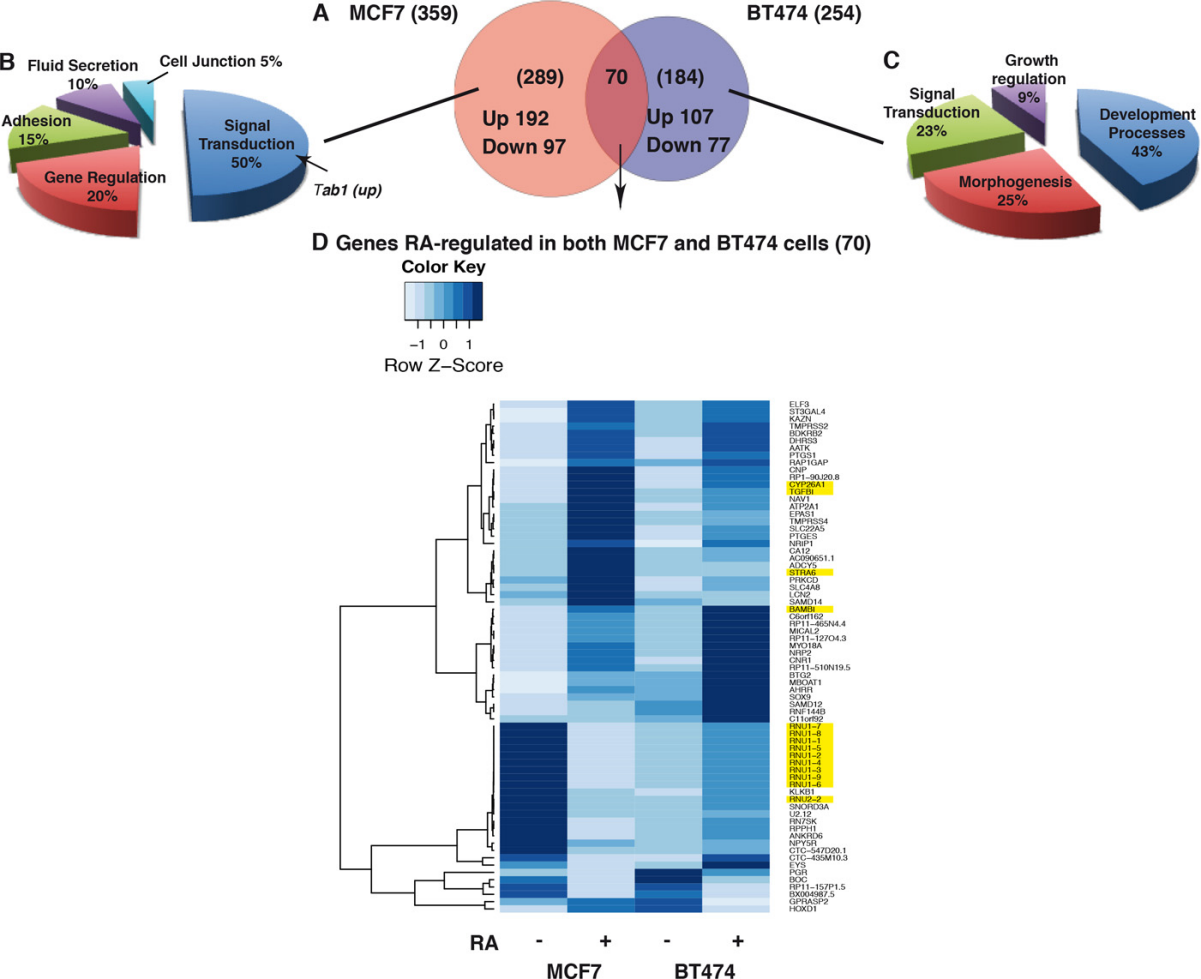
Comparison of the RA-regulated genes in MCF7 and BT474 cells. (A) Venn diagram showing that 80% of the genes that are RA-regulated in MCF7 cells are not in BT474 cells. (B) and (C) 3D pie charts showing the categories of genes that are RA-regulated in MCF7 only and in BT474 cells only. The genes were selected using the Manteia GO statistical analysis on GO analysis with a P value <0,01. (D) Heatmaps showing the genes that are RA-regulated in both cell lines.

Interestingly, MCF7 cells depicted 40% more RA-regulated genes than BT474 cells. The Venn diagram in Fig 12A shows that 80% of the RA-regulated genes in MCF7 cells were not regulated in BT474 cells and reciprocally. According to enriched Gene Ontology (GO), these genes do not belong to the same functional groups (Fig 12B and 12C).

In fact, 70 genes corresponding to 20% of the RA-regulated genes in MCF7 cells were also RA-regulated in BT474 cells. However according to the Heatmap in Fig 12D, genes that were up regulated by RA in MCF7 cells were down regulated in BT474 cells. Reciprocally, other genes exemplified by a group of U1 small nuclear RNAs (RNU1) were up regulated in BT474 cells and down regulated in MCF7 cells. Remarkably, a subset of genes (30%) exemplified by the canonical RA target genes *Cyp26a1* (cytochrome P450, family 26, subfamily b, polypeptide 1), *Stra6* (stimulated by retinoic acid gene 6 homolog) and *TGF1β* (transforming growth factor, beta-induced), were less efficiently up regulated by RA in BT474 than in MCF7 cells. The opposite was found for an other group (20%) exemplified by the *BAMBI* (BMP And Activin Membrane-Bound Inhibitor) gene, which functions as a negative regulator of the TGF1β signaling pathway [38]. Thus components of the TGF1β pathway are induced in RA sensitive cells [39] and repressed in RA resistant ones. Corroborating this conclusion, the *TAB1* gene, which encodes an intermediary protein between the TGFβ receptor and MAPKs [40] was induced in MCF7 cells only (Fig 12B). Altogether these results suggest that RA resistance would reflect a deregulation of most of the RA-target genes, including genes encoding components of the TGF1β pathway.

### In RA-resistant BT474 cells, RARα is less efficiently recruited to gene promoters

The expression of the RARα-target genes is controlled by the RA-induced recruitment of RARα to specific response elements (RAREs) located in the promoters of the target genes. As this recruitment requires the phosphorylation of RARα at S77 [20, 21], one can propose that the deregulation observed in BT474 cells would reflect the absence of recruitment of RARα subsequent to its deficient phosphorylation.

**Fig 13:**
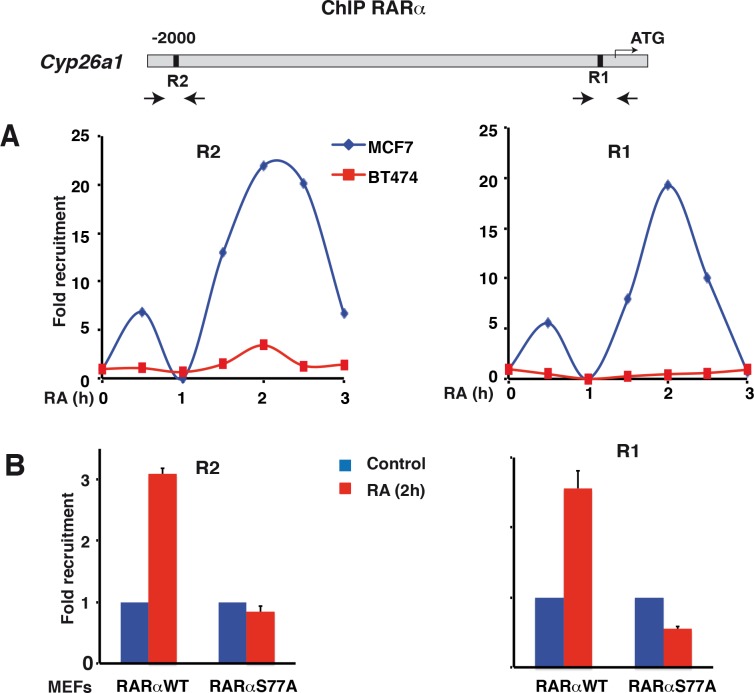
ChIP-qPCR analysis of RARα recruitment at the *Cy26a1* gene promoter. (A) Kinetic ChIP experiments performed with RA-treated MCF7 and BT474 cells and determining the recruitment of RARα to the R1 and R2 response elements of the *Cyp26a1* gene. Values correspond to a representative experiment among 3. (B) ChIP experiments performed with MEFs expressing RARαWT or RARαS77A and determining the recruitment of RARα to the R1 and R2 elements of the *Cyp26a1* gene. Values are the mean ±SD of three experiments.

Therefore, we analyzed the recruitment of RARα to the promoter of the canonical *Cyp26a1* gene which is less efficiently up-regulated by RA in BT474 cells, compared to MCF7 cells (Fig 12D) [22]. ChIP-qPCR experiments showed that in MCF7 cells, RARα was rapidly recruited to both the proximal (R1) and distal (R2) RAREs located in the promoter of the *Cyp26A1* gene (Fig 13A). However RARα recruitment to these elements was markedly decreased in BT474 cells (Fig 13A). It is worth noting that in MEFs expressing RARαS77A, which is phosphorylated neither at S77 nor at S74, RARα was not recruited either at the R1 and R2 elements of the *Cyp26a1* gene promoter (Fig 13B). Collectively, these results indicate that the differential gene regulation observed in the RA-resistant BT474 cells might be correlated at least in part to a deficient phosphorylation and DNA recruitment of RARα.

## Discussion

The aim of the present study was to compare the phosphoproteome of RA sensitive and RA resistant breast cancer cells and to determine whether a deregulated kinome impacts the RA response. We selected two human breast cancer cell lines, the MCF7 and the BT474 cell lines, which are responsive and resistant to RA respectively. BT474 cells depict in addition to several PI3K mutations, ERB-B2 amplification with deregulation of the downstream kinases [14, 41] and thus are a model of choice to analyze the consequences of a deregulated kinome on the RA response in terms of phosphorylation cascades and of gene regulation.

We compared the two cell lines in a large-scale analysis of the phosphorylated proteins and in a genome wide analysis of the RA-regulated genes. The interesting conclusion of this study is that MCF7 and BT474 cells show differences in (i) their phosphoprotein profiles, (ii) their RA-regulated phosphoproteins profiles and (iii) in their RA-regulated genes profiles.

We performed the first MS study of the phosphoproteome and of the phosphorylation events induced by RA in these two cell lines, taking advantage of the recent Orbitrap technology combined to phosphopeptides enrichment. The analysis of the results revealed that several cytosolic and nuclear proteins are phosphorylated in both cell lines. The former are essentially involved in signal transduction, RNA translation and adhesion while the latter are involved in transcription and in chromatin modifications/organization.

The first novelty of the present study is the observation that several proteins were phosphorylated in MCF7 cells only and others in BT474 cells only (Fig 5 and Fig 7). Moreover, RA did not regulate the phosphorylation of the same proteins in the two cell lines, indicating that BT474 cells depict a deregulated kinome, compared to MCF7 cells.

We also performed the first MS analysis of the RARα phosphorylation sites after several technical developments. Indeed, we successfully produced antibodies, which efficiently enriched RARα by immunoprecipitation. We also selected thermolysin, which led to high sequence coverage and generated properly sized peptides embedding the phosphorylation sites. The novelty is the finding that, in MCF7 cells, RA induces the phosphorylation of two serine residues (S77 and S74) located in the NTD. The other novelty is that RA does not induce the phosphorylation of residues in BT474 cells. Given that Herceptin partially restores their phosphorylation, one can speculate that this deregulations reflects, at least in part, the overexpression and phosphorylation of the receptor tyrosine kinase ERB-B2, which activates the downstream PI3K/Akt pathway.

The other novelty is the observation, in a genome wide analysis of the RA-regulated genes, that, in BT474 cells, several canonical RA-target genes were less efficiently up regulated than in MCF7 cells. As the expression of these genes requires the recruitment of the phosphorylated form of RARα at promoters [20, 21], one can speculate that the observed deficient regulation would reflect the absence of phosphorylation of RARα. However the opposite was observed for other genes. In addition, most of the genes that were regulated in MCF7 cells were not in BT474 cells and reciprocally. Thus one cannot exclude that that such discrepancies reflect not only the deficient phosphorylation of RARα but also alterations in the phosphorylation of other transcriptions factors and of chromatin or DNA modifiers (see Fig 7). Studying how the phosphorylation of such factors controls the expression of these genes is out of the scope of this manuscript.

Most interestingly, our results indicate that in MCF7 cells only, RA increases the expression of *TGF1β* and induces the expression of its downstream target *TAB1*, which are both involved in the activation of MAPKs. They also indicate that in BT474 cells only, RA increases the expression of *BAMBI*, which functions as a negative regulator of the TGF1β signaling pathway [38]. Thus, in the RA resistant BT474 cells, not only the phosphorylation state but also the expression levels of the signaling proteins are altered.

In conclusion, this study highlights the importance of the integrity of the kinome for the ability of RA to activate kinases with consequences on the phosphorylation of RARα and several other nuclear proteins involved in transcription and thus on gene expression. Considering that phosphorylation processes control either positively or negatively DNA binding and protein-protein interactions and are part of RA action [20, 21, 42], this study opens new avenues in the understanding of the deregulation of the RA response in cancer or other diseases.

## Supporting Information

S1 Fig Purity test of the cytosolic and nuclear extracts prepared from MCF7 and BT474 cells. An aliquot of each extract was immunoblotted with β-tubulin and lamin A/C antibodies. β-tubulin is present exclusively in the cytosol and lamin A/C exclusively in the nucleus
(TIF)

S2 Fig Phosphorylation motifs. Twenty-five motifs were significantly represented in the cytosol of the vehicle-treated MCF7 cells in experiment R1, using the Motif-X algorithm. (A) Distribution of the different motifs in the dataset. Three percent of the phosphorylation sites did not lead to any motif attribution according to the defined significance criteria. (B) Sequence logos of the various motifs. (C) The motifs are shown with their respective score and their occurrence in the dataset (Foreground Matches) and in the IPI Human Proteome (Background Matches). The relative enrichment of the motifs in the dataset compared to the IPI Human Proteome is also shown (Fold Increase). Similar results were obtained for the other cytosolic or nuclear samples.
(TIF)

S3 Fig **Number of phosphosites per protein, in MCF7 (A) and in BT 474 cells (B).** The results were obtained from the cytosolic extracts (C) and nuclear extracts (N) in experiment 1
(TIF)

S1 Table List of the proteins and phosphoproteins identified in the nuclear and cytoplasmic extracts of MCF7 and BT474 cells with and without RA treatment in the first replicate experiment R1. Only the proteins identified with at least two peptides with high confidence in both the control- and RA-treated extracts were selected.
(XLSX)

S2 Table Same as S1 Table for the second replicate experiment R2. (XLSX)

S3 Table Description of the phosphorylated peptides and of the phosphosites grouped per protein in the replicate experiment R1. (XLSX)

S4 Table Description of the phosphorylated peptides and of the phosphosites grouped per protein in the replicate experiment R2. (XLSX)

S5 Table Description of the RARα phosphorylated peptides identified in MCF7 and BT474 cells. The presented data correspond to a representative experiment among two.
(XLSX)

S6 Table List of the genes that are regulated by RA in MCF7 and BT474 cells. Ensembl IDs, gene names, descriptions and normalized expression values for transcripts that are induced or repressed by RA in the different cell lines are shown. The log2 change in expression and adjusted p value are also indicated.
(XLS)

## References

[1] Rochette-Egly C, Germain P. Dynamic and combinatorial control of gene expression by nuclear retinoic acid receptors. Nuclear Receptor Signaling. 2009;7:e005 10.1621/nrs.07005 19471584 PMC2686084

[2] Samarut E, Rochette-Egly C. Nuclear retinoic acid receptors: conductors of the retinoic acid symphony during development. Mol Cell Endocrinol. 2012;348(2):348–60. Epub 2011/04/21. 10.1016/j.mce.2011.03.025 . 21504779

[3] Moutier E, Ye T, Choukrallah MA, Urban S, Osz J, Chatagnon A, et al Retinoic acid receptors recognise the mouse genome through binding elements with diverse spacing and topology. J Biol Chem. 2012;287(31):26328–41. Epub 2012/06/05. 10.1074/jbc.M112.361790 . 22661711 PMC3406717

[4] Carroll JS, Meyer CA, Song J, Li W, Geistlinger TR, Eeckhoute J, et al Genome-wide analysis of estrogen receptor binding sites. Nat Genet. 2006;38(11):1289–97. Epub 2006/10/03. 10.1038/ng1901 . 17013392

[5] Hua S, Kittler R, White KP. Genomic antagonism between retinoic acid and estrogen signaling in breast cancer. Cell. 2009;137(7):1259–71. Epub 2009/07/01. 10.1016/j.cell.2009.04.043 . 19563758 PMC3374131

[6] Mahony S, Mazzoni EO, McCuine S, Young RA, Wichterle H, Gifford DK. Ligand-dependent dynamics of retinoic acid receptor binding during early neurogenesis. Genome Biol. 2011;12(1):R2 Epub 2011/01/15. 10.1186/gb-2011-12-1-r2 21232103 PMC3091300

[7] Mendoza-Parra MA, Walia M, Sankar M, Gronemeyer H. Dissecting the retinoid-induced differentiation of F9 embryonal stem cells by integrative genomics. Mol Syst Biol. 2011;7:538 Epub 2011/10/13. 10.1038/msb.2011.73 21988834 PMC3261707

[8] Al Tanoury Z, Piskunov A, Rochette-Egly C. Vitamin A and retinoid signaling: genomic and nongenomic effects: Thematic Review Series: Fat-Soluble Vitamins: Vitamin A. J Lipid Res. 2013;54(7):1761–75. Epub 2013/02/27. 10.1194/jlr.R030833 23440512 PMC3679380

[9] Piskunov A, Rochette-Egly C. A retinoic acid receptor RARalpha pool present in membrane lipid rafts forms complexes with G protein alphaQ to activate p38MAPK. Oncogene. 2012;31(28):3333–45. Epub 2011/11/08. 10.1038/onc.2011.499 . 22056876

[10] Piskunov A, Al Tanoury Z, Rochette-Egly C. Nuclear and extra-nuclear effects of retinoid acid receptors: how they are interconnected. Sub-cellular biochemistry. 2014;70:103–27. 10.1007/978-94-017-9050-5_6 . 24962883

[11] Lalevee S, Ferry C, Rochette-Egly C. Phosphorylation control of nuclear receptors. Methods in molecular biology. 2010;647:251–66. 10.1007/978-1-60761-738-9_15 . 20694672

[12] Duong V, Rochette-Egly C. The molecular physiology of nuclear retinoic acid receptors. From health to disease. Biochim Biophys Acta. 2011;1812(8):1023–31. Epub 2010/10/26. 10.1016/j.bbadis.2010.10.007 . 20970498

[13] Blume-Jensen P, Hunter T. Oncogenic kinase signalling. Nature. 2001;411(6835):355–65. 10.1038/35077225 . 11357143

[14] She QB, Chandarlapaty S, Ye Q, Lobo J, Haskell KM, Leander KR, et al Breast tumor cells with PI3K mutation or HER2 amplification are selectively addicted to Akt signaling. PloS one. 2008;3(8):e3065 10.1371/journal.pone.0003065 18725974 PMC2516933

[15] Tari AM, Lim SJ, Hung MC, Esteva FJ, Lopez-Berestein G. Her2/neu induces all-trans retinoic acid (ATRA) resistance in breast cancer cells. Oncogene. 2002;21(34):5224–32. 10.1038/sj.onc.1205660 . 12149644

[16] Jones KA, Kim PD, Patel BB, Kelsen SG, Braverman A, Swinton DJ, et al Immunodepletion plasma proteomics by tripleTOF 5600 and Orbitrap elite/LTQ-Orbitrap Velos/Q exactive mass spectrometers. Journal of proteome research. 2013;12(10):4351–65. 10.1021/pr400307u 24004147 PMC3817719

[17] Kalli A, Smith GT, Sweredoski MJ, Hess S. Evaluation and optimization of mass spectrometric settings during data-dependent acquisition mode: focus on LTQ-Orbitrap mass analyzers. Journal of proteome research. 2013;12(7):3071–86. Epub 2013 May 31. 10.1021/pr3011588 23642296 PMC3748959

[18] Engholm-Keller K, Larsen MR. Titanium dioxide as chemo-affinity chromatographic sorbent of biomolecular compounds—applications in acidic modification-specific proteomics. Journal of proteomics. 2011;75(2):317–28. Epub 2011 Aug 4. 10.1016/j.jprot.2011.07.024 . 21840430

[19] Leitner A, Sturm M, Lindner W. Tools for analyzing the phosphoproteome and other phosphorylated biomolecules: a review. Analytica chimica acta. 2011;703(1):19–30. Epub 2011 Aug 4. 10.1016/j.aca.2011.07.012 . 21843671

[20] Al Tanoury Z, Gaouar S, Piskunov A, Ye T, Urban S, Jost B, et al Phosphorylation of the retinoic acid receptor RARgamma2 is crucial for the neuronal differentiation of mouse embryonic stem cells. J Cell Sci. 2014;147:2095–105. Epub 2014/02/27. 10.1242/jcs.145979 . 24569880

[21] Bruck N, Vitoux D, Ferry C, Duong V, Bauer A, de The H, et al A coordinated phosphorylation cascade initiated by p38MAPK/MSK1 directs RARalpha to target promoters. Embo J. 2009;28(1):34–47. Epub 2008 Dec 11. 10.1038/emboj.2008.256 19078967 PMC2633082

[22] Ferry C, Gaouar S, Fischer B, Boeglin M, Paul N, Samarut E, et al Cullin 3 mediates SRC-3 ubiquitination and degradation to control the retinoic acid response. Proceedings of the National Academy of Sciences of the United States of America. 2011;108(51):20603–8. Epub 2011 Dec 6. 10.1073/pnas.1102572108 22147914 PMC3251120

[23] Gaub MP, Rochette-Egly C, Lutz Y, Ali S, Matthes H, Scheuer I, et al Immunodetection of multiple species of retinoic acid receptor alpha: evidence for phosphorylation. Experimental cell research. 1992;201(2):335–46. . 1322315 10.1016/0014-4827(92)90282-d

[24] Al Tanoury Z, Piskunov A, Andriamoratsiresy D, Gaouar S, Lutzing R, Ye T, et al Genes involved in cell adhesion and signaling: a new repertoire of retinoic acid receptor target genes in mouse embryonic fibroblasts. J Cell Sci. 2014;127(Pt 3):521–33. Epub 2013 Dec 19. 10.1242/jcs.131946 . 24357724

[25] Trapnell C, Pachter L, Salzberg SL. TopHat: discovering splice junctions with RNA-Seq. Bioinformatics. 2009;25(9):1105–11. Epub 2009/03/18. 10.1093/bioinformatics/btp120 19289445 PMC2672628

[26] Trapnell C, Williams BA, Pertea G, Mortazavi A, Kwan G, van Baren MJ, et al Transcript assembly and quantification by RNA-Seq reveals unannotated transcripts and isoform switching during cell differentiation. Nature biotechnology. 2010;28(5):511–5. Epub 2010 May 2. 10.1038/nbt.1621 20436464 PMC3146043

[27] Anders S, Huber W. Differential expression analysis for sequence count data. Genome Biol. 2010;11(10):R106 Epub 2010/10/29. 10.1186/gb-2010-11-10-r106 . 20979621 PMC3218662

[28] Benjamini Y, Hochberg Y. Controlling the false discovery rate: a practical and powerful approach to multiple testing. Journal of the Royal Statistical Society Series. 1995;57(B):289–300.

[29] Tassy O, Pourquie O. Manteia, a predictive data mining system for vertebrate genes and its applications to human genetic diseases. Nucleic acids research. 2014;42(Database issue):D882–91. Epub 2013 Sep 12. 10.1093/nar/gkt807. 10.1093/nar/gkt807 24038354 PMC3964984

[30] Bour G, Plassat JL, Bauer A, Lalevee S, Rochette-Egly C. Vinexin beta interacts with the non-phosphorylated AF-1 domain of retinoid receptor gamma (RARgamma) and represses RARgamma-mediated transcription. J Biol Chem. 2005;280(17):17027–37. Epub 2005 Feb 25. 10.1074/jbc.M501344200 . 15734736

[31] Dickhut C, Feldmann I, Lambert J, Zahedi RP. Impact of digestion conditions on phosphoproteomics. Journal of proteome research. 2014;13(6):2761–70. Epub 2014 May 7. 10.1021/pr401181y . 24724590

[32] Huttlin EL, Jedrychowski MP, Elias JE, Goswami T, Rad R, Beausoleil SA, et al A tissue-specific atlas of mouse protein phosphorylation and expression. Cell. 2010;143(7):1174–89. 10.1016/j.cell.2010.12.001 21183079 PMC3035969

[33] Olsen JV, Blagoev B, Gnad F, Macek B, Kumar C, Mortensen P, et al Global, in vivo, and site-specific phosphorylation dynamics in signaling networks. Cell. 2006;127(3):635–48. 10.1016/j.cell.2006.09.026 . 17081983

[34] Ma X, Zhao H, Shan J, Long F, Chen Y, Chen Y, et al PDCD10 interacts with Ste20-related kinase MST4 to promote cell growth and transformation via modulation of the ERK pathway. Mol Biol Cell. 2007;18(6):1965–78. Epub 2007 Mar 14. 10.1091/mbc.E06-07-0608 17360971 PMC1877091

[35] Thompson BJ, Sahai E. MST kinases in development and disease. J Cell Biol. 2015;210(6):871–82. 10.1083/jcb.201507005 26370497 PMC4576864

[36] Kim BR, Seo SH, Park MS, Lee SH, Kwon Y, Rho SB. sMEK1 inhibits endothelial cell proliferation by attenuating VEGFR-2-dependent-Akt/eNOS/HIF-1alpha signaling pathways. Oncotarget. 2015;6(31):31830–43. 10.18632/oncotarget.5570 26378810 PMC4741643

[37] Rochette-Egly C, Adam S, Rossignol M, Egly JM, Chambon P. Stimulation of RAR alpha activation function AF-1 through binding to the general transcription factor TFIIH and phosphorylation by CDK7. Cell. 1997;90(1):97–107. Epub 1997/07/11. . 9230306 10.1016/s0092-8674(00)80317-7

[38] Yan X, Lin Z, Chen F, Zhao X, Chen H, Ning Y, et al Human BAMBI cooperates with Smad7 to inhibit transforming growth factor-beta signaling. J Biol Chem. 2009;284(44):30097–104. Epub 2009 Sep 15. 10.1074/jbc.M109.049304 19758997 PMC2781564

[39] Fadloun A, Kobi D, Delacroix L, Dembele D, Michel I, Lardenois A, et al Retinoic acid induces TGFbeta-dependent autocrine fibroblast growth. Oncogene. 2008;27(4):477–89. Epub 2007 Jul 16. 10.1038/sj.onc.1210657 . 17637747

[40] Shibuya H, Yamaguchi K, Shirakabe K, Tonegawa A, Gotoh Y, Ueno N, et al TAB1: an activator of the TAK1 MAPKKK in TGF-beta signal transduction. Science. 1996;272(5265):1179–82. . 8638164 10.1126/science.272.5265.1179

[41] Ali NA, Wu J, Hochgrafe F, Chan H, Nair R, Ye S, et al Profiling the tyrosine phosphoproteome of different mouse mammary tumour models reveals distinct, model-specific signalling networks and conserved oncogenic pathways. Breast cancer research: BCR. 2014;16(5):437 10.1186/s13058-014-0437-3 25200860 PMC4303118

[42] Bour G, Lalevee S, Rochette-Egly C. Protein kinases and the proteasome join in the combinatorial control of transcription by nuclear retinoic acid receptors. Trends in cell biology. 2007;17(6):302–9. Epub 2007 Apr 30. 10.1016/j.tcb.2007.04.003 . 17467991

