# Supplementary figures and images for: Phosphoproteome and Transcriptome of RA-Responsive and RA-Resistant Breast Cancer Cell Lines
Source: PLoS One. 2016 Jun 30;11(6):e0157290. doi: 10.1371/journal.pone.0157290 (PMC4928811; doi:10.1371/journal.pone.0157290)

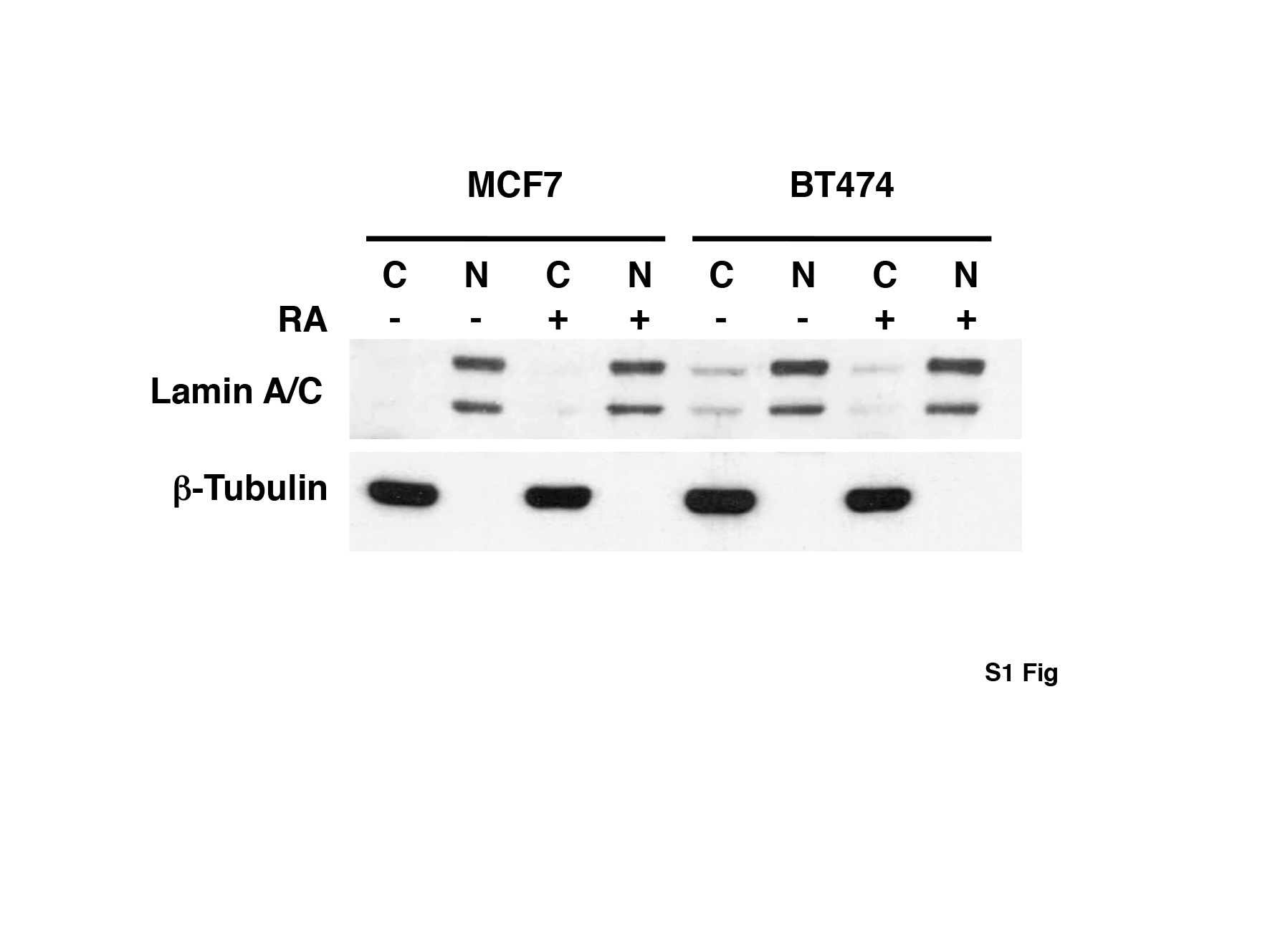

Supplement: S1 Fig — An aliquot of each extract was immunoblotted with β-tubulin and lamin A/C antibodies. β-tubulin is present exclusively in the cytosol and lamin A/C exclusively in the nucleus (TIF) [file pone.0157290.s001.tif]

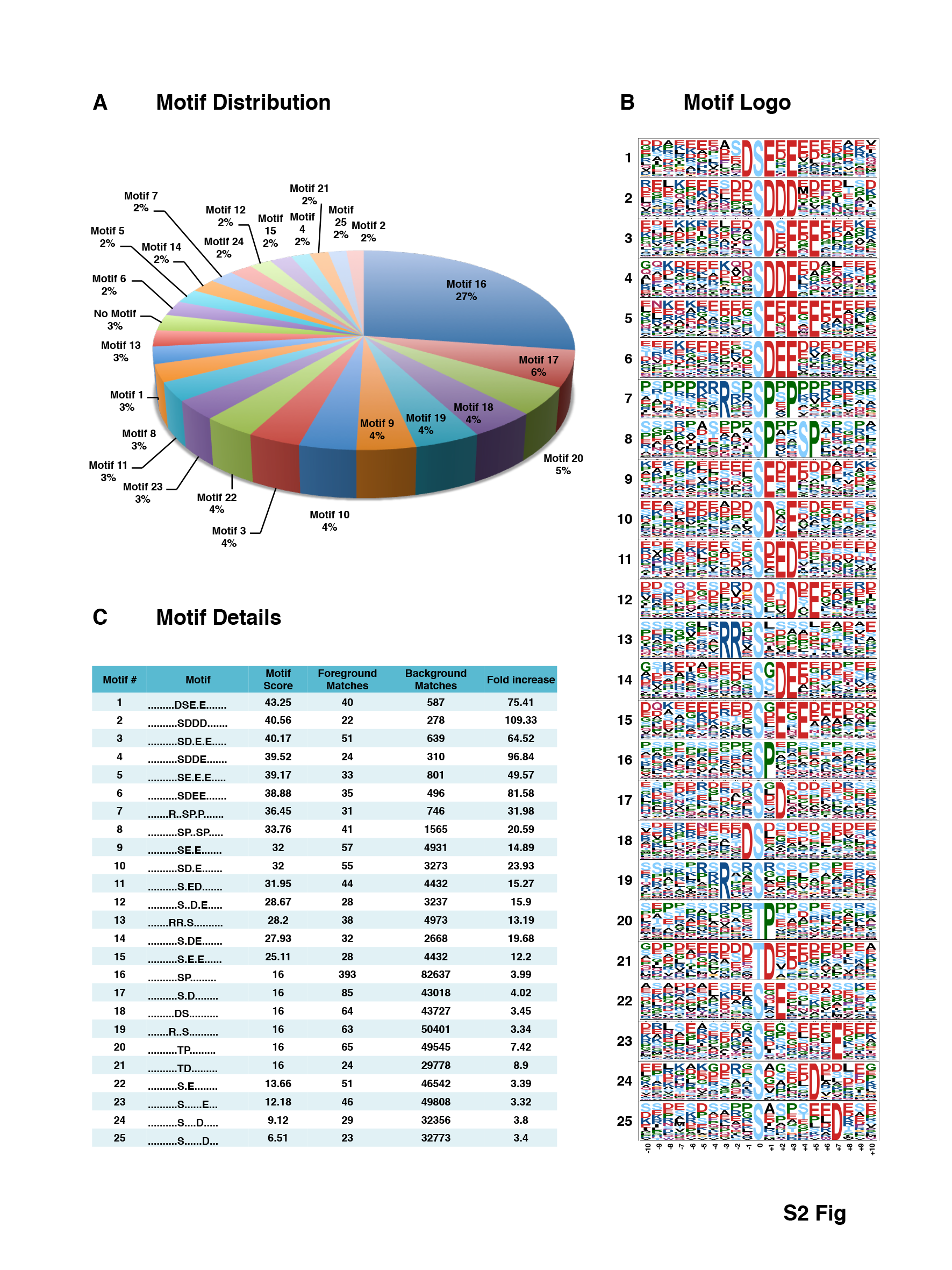

Supplement: S2 Fig — Twenty-five motifs were significantly represented in the cytosol of the vehicle-treated MCF7 cells in experiment R1, using the Motif-X algorithm. (A) Distribution of the different motifs in the dataset. Three percent of the phosphorylation sites did not lead to any motif attribution according to the defined significance criteria. (B) Sequence logos of the various motifs. (C) The motifs are shown with their respective score and their occurrence in the dataset (Foreground Matches) and in the IPI Human Proteome (Background Matches). The relative enrichment of the motifs in the dataset compared to the IPI Human Proteome is also shown (Fold Increase). Similar results were obtained for the other cytosolic or nuclear samples. (TIF) [file pone.0157290.s002.tif]

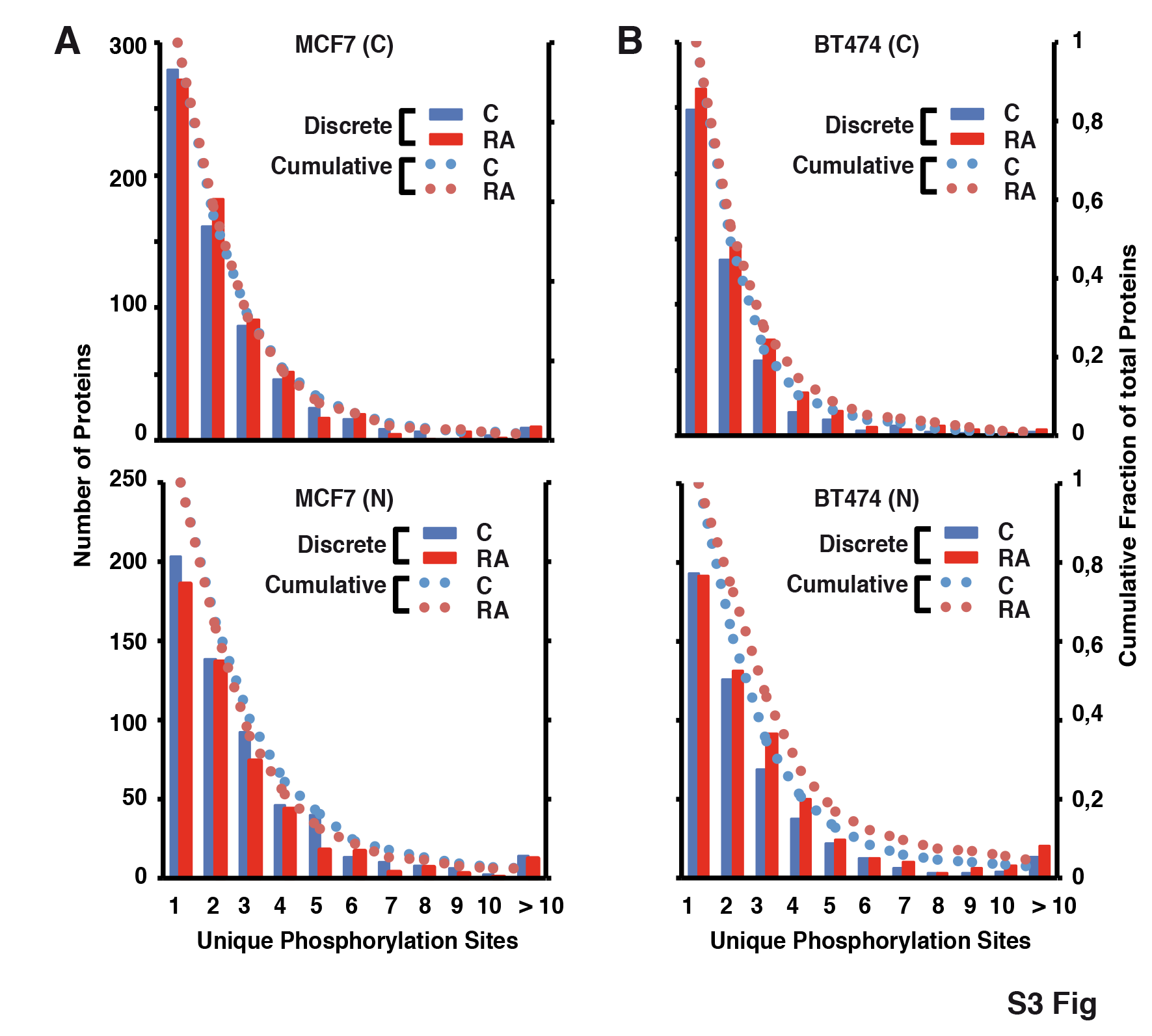

Supplement: S3 Fig — Number of phosphosites per protein, in MCF7 (A) and in BT 474 cells (B). The results were obtained from the cytosolic extracts (C) and nuclear extracts (N) in experiment 1 (TIF) [file pone.0157290.s003.tif]
